# Unveiling a Disease Complex Threatening Fig (*Ficus carica* L.) Cultivation in Southern Italy

**DOI:** 10.3390/plants14182865

**Published:** 2025-09-15

**Authors:** Wassim Habib, Mariangela Carlucci, Vincenzo Cavalieri, Cecilia Carbotti, Franco Nigro

**Affiliations:** 1 Centro di Ricerca, Formazione e Sperimentazione in Agricoltura—Basile Caramia (CRSFA), Via Cisternino 281, 70010 Locorotondo, Italy; wassimhabib@crsfa.itvincenzo.cavalieri@ipsp.cnr.it (V.C.); 2 Dipartimento di Scienze del Suolo, della Pianta e degli Alimenti, Università degli Studi di Bari—Aldo Moro, Via Amendola 165/A, 70126 Bari, Italy; mariangela.carlucci@uniba.it (M.C.); c.carbotti6@phd.uniba.it (C.C.); 3 Istituto per la Protezione Sostenibile delle Piante—CNR, Via Amendola 122/D, 70126 Bari, Italy

**Keywords:** *Ficus carica*, *Ceratocystis ficicola*, *Neofusicoccum* spp., *Neocosmospora* spp., *Cryphalus dilutus*

## Abstract

Fig (*Ficus carica*) orchards in the Salento peninsula (southeastern Apulia region, Italy) are increasingly affected by decline syndromes whose etiology remains poorly resolved. In this paper, we provide a first characterization of a complex disease outbreak, integrating field surveys, fungal isolation, molecular phylogenetics, and pathogenicity assays. Symptomatic trees displayed chlorosis, defoliation, cankers, vascular discoloration, and wilting, frequently associated with bark beetle galleries. Mycological analyses revealed a diverse assemblage of fungi, dominated by Botryosphaeriaceae (including *Neofusicoccum algeriense*, and *Lasiodiplodia theobromae*), the *Fusarium solani* species complex (notably *Neocosmospora perseae*), and *Ceratocystis ficicola*. While *C. ficicola* was isolated with lower frequency, its recovery from adult beetles—including *Cryphalus dilutus*—supports a role in insect-mediated dissemination in addition to soilborne infection. Pathogenicity tests demonstrated that *N. algeriense* and *N. perseae*, together with *C. ficicola*, caused severe vascular lesions and wilting, confirming their contribution to fig decline. By contrast, other Fusarioid strains showed no pathogenicity, consistent with their role as latent or stress-associated pathogens. This study provides the first evidence that *N. algeriense* and *N. perseae* act as pathogenic agents on fig, highlights their interaction with *C. ficicola* within a multifactorial decline syndrome, and identifies dual epidemiological pathways involving both soil/root infection and insect-facilitated dissemination via beetles such as *C. dilutus*. These findings redefine fig decline in the Salento peninsula (southern Italy) as a multifactorial disease rather than a single-pathogen outbreak, with significant implications for diagnosis, epidemiology, and integrated management strategies.

## 1. Introduction

The common fig (*Ficus carica* L.) is considered one of the earliest domesticated plants, as evidenced by archeobotanical findings of parthenocarpic fruits dating back to the 12th millennium BCE [1,2]. Native to regions such as Persia, Asia Minor, and Syria, the fig tree now grows wild or feral across most Mediterranean countries [3]. Its historical significance is underscored by numerous references in both the Bible and the Quran, and its cultivation was widespread during the Roman Empire.

The fig is among the most economically significant fruit trees, and is cultivated in both open fields and greenhouses. Approximately 70% of global fig production occurs in Mediterranean countries, where it plays a vital role in the regional diet [4].

In Italy, fig cultivation spans an area of 2071 hectares, with 500 hectares located in the Apulia region (southeastern Italy), yielding an annual production of 3200 tons [5]. Apulia hosts a diverse germplasm of edible figs and caprifigs (the wild male form of the common fig, producing inedible fruits that serve as pollen and wasp hosts for pollination), likely originating from various eastern regions of the Roman Empire, making it a valuable repository of fig genetic diversity.

Figs are susceptible to numerous parasites and pathogens that can cause severe diseases. Approximately 77 species of scale insects from the families Diaspididae, Pseudococcidae, and Coccidae are known to attack *Ficus carica* worldwide, though information on pests affecting fig cultivation in Italy remains limited [6]. Recently, new pests such as the black weevil *Aclees taiwanensis* Kono, the bark beetle *Cryphalus dilutus* (Eichhoff) (previously identified as *Hypocryphalus scabricollis* (Eichhoff)), and the ambrosia beetle *Xyleborus bispinatus* Eichhoff have been reported on figs in Italy.

*A. taiwanensis*, a major threat to fig trees, was first recorded in France (1997) and later in Italy (2005), with occurrences reported in several northern and central Italian regions and one region in France [7]. *C. dilutus* was initially recorded in Malta (1991), and along with *X. bispinatus* was reported in Sicily in 2014 [8]. These pests are linked to the rapid decline in and desiccation of fig trees across diverse settings, including individual trees, small groups, and large plantations [8].

Fungal pathogens, particularly those from the Botryosphaeriaceae family, are significant contributors to canker disease in figs worldwide. Members of this family are globally distributed and affect a wide range of hosts, including monocotyledons, dicotyledons, gymnosperms, and angiosperms, functioning as saprotrophs, parasites, or endophytes [9,10]. In Italy, notable hosts include olive (*Olea europaea* L.), grapevine (*Vitis vinifera* L), and common fig [11,12,13,14].

Key pathogenic genera such as *Diplodia*, *Lasiodiplodia*, and *Neofusicoccum* cause a variety of symptoms, including dieback, cankers, blight, and rot on aboveground plant organs [9]. Examples include *Botryosphaeria dothidea*, causing bot rot on figs in China [15]; *Lasiodiplodia theobromae*, reported on figs in Turkey [16], China, and South Korea [17,18]; *Neofusicoccum parvum*, associated with branch canker and stem-end fruit rot on avocados (*Persea americana* Mill) [19], mangoes (*Mangifera indica* L.) [20], and figs in Sicily, Italy [14]; and *Neoscytalidium dimidiatum*, identified on figs in Oman [21], Australia [22], and California [23].

The genus *Fusarium* comprises at least 20 species complexes, including isolates capable of causing plant diseases. The *Fusarium oxysporum* species complex (FOSC) includes soilborne filamentous fungi with a broad host range, affecting plants and animals. In agriculture, *F. oxysporum* is a major pathogen, causing significant economic losses in crops such as tomatoes, bananas, legumes, and cotton [24].

Similarly, the *Fusarium solani* species complex (FSSC) contains many economically significant phytopathogenic species. Within this complex, the genus *Neocosmospora* (previously part of the *F. solani* species) now encompasses saprobes, plant endophytes, and significant plant pathogens, as well as opportunistic animal pathogens [25,26]. It also includes tree-pathogenic mutualists of the shot-hole borer beetle (*Euwallacea* spp.), associated with dieback in avocado and tea (*Camelia sinensis* L.) [27,28].

In Italy, several *Neocosmospora* species have been identified as pathogens on fruit crops, including citrus, figs, avocados, and raspberries (*Rubus idaeus* L.) [26,29,30,31]. Recently, *N. metavorans* and a newly described species, *N. caricae*, were reported as stem and trunk canker pathogens of *Ficus carica* in Iran [32].

Ophiostomatoid fungi, members of the class *Sordariomycetes* within the phylum *Ascomycota*, belong to the orders *Ophiostomatales* (Sordariomycetidae) and *Microascales* (Hypocreomycetidae) [33]. The genus *Ceratocystis*, within the family Ceratocystidaceae (*Microascales*), includes pathogens responsible for significant tree diseases worldwide [34]. Many species in this genus are closely associated with scolytid beetles, which serve as key vectors [35]. For instance, *Scolytus scolytus* and *S. multistriatus* effectively transmit *Ophiostoma ulmi* and *O. novo-ulmi*, causal agents of Dutch elm disease [36,37,38].

The bark beetle *C. dilutus* (Eichhoff), previously identified as *Hypocryphalus mangifera* (Stebbing), vectors mango sudden death disease caused by *C. fimbriata* sensu lato [39]. Initially reported on figs in Italy in 2014 as *Hypocryphalus scabricollis* (Eichhoff) [8], this beetle was reclassified as *C. dilutus* in 2018 by Johnson et al. [40]. In recent years, *C. dilutus* has been associated with fig decline in Apulia, Italy, linked to fungal pathogens [41]. It was also identified as an invasive species in Europe, being associated with wood infections in mango trees in Sicily, particularly involving *C. ficicola* Kajitani & Masuya [42].

*Ceratocystis ficicola*, a severe pathogen of *Ficus carica*, was first reported in Japan [43] and later in Greece [44]. This pathogen has been documented on figs in Apulia [45] and Sicily [46], highlighting its expanding geographic and host range. Affected fig trees initially display leaf chlorosis, which gradually progresses to necrosis, twig defoliation, and dieback of lateral branches. Between 2021 and 2025, severe decline symptoms were observed in *Ficus carica* in the Salento area of the Apulia region (latitude: 39.9500° N to 40.5500° N; longitude: 17.5000° E to 18.5000° E), with a preliminary account previously reported by Habib et al. [45].

The objectives of this study were as follows.

(i) Identify the fungal pathogens associated with fig decline symptoms in southern Apulia (southeastern Italy) using a multilocus phylogenetic approach to achieve accurate species-level identification.

(ii) Evaluate the pathogenicity of the identified fungal species on fig seedlings under controlled conditions, providing insights into their roles in disease progression.

(iii) Identify the insect species recovered from infected host tissues, with a focus on their potential roles as vectors or facilitators of fungal dissemination.

## 2. Results

### 2.1. Field Survey, Fungal Isolation, and Morphological Characterization of the Isolates

**Figure 1:**
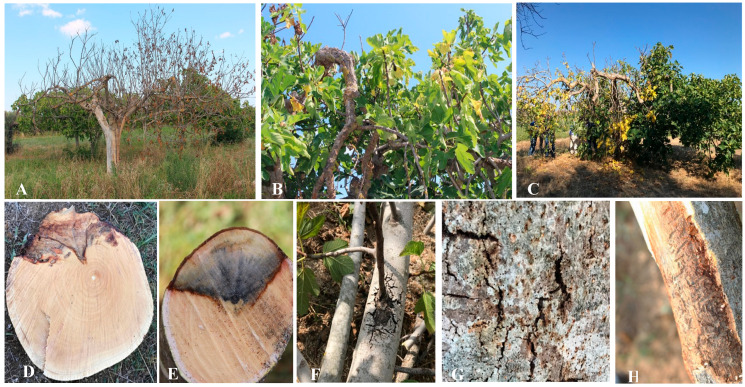
Field images of infected fig trees (**A**); infected mature tree with symptoms of leaf wilt and leaf chlorosis (**B**); necrosis, twig defoliation, and death of lateral branches (**C**); extensive wood dis-coloration under the dead bark (**D**,**E**). Cankers on the lateral branches (**F**); bark beetles’ holes (**G**) and galleries under the bark (**H**).

The disease was recorded in long-established fig orchards, with trees ranging in age from 5 to 50 years and cultivated in open fields across the Salento area of Apulia. A consistent set of external and internal symptoms was documented on affected fig trees. Initial signs included foliar chlorosis progressing to necrosis, followed by premature defoliation and dieback of lateral branches (Figure 1). Cankers were frequently observed in association with extensive internal wood discoloration beneath dead bark, often coinciding with bark beetle galleries (Figure 1). Fruit quality was also reduced, with visible rot and loss of marketability. As the disease progressed, wilting intensified, frequently leading to complete canopy defoliation, where premature leaf drop severely impaired photosynthetic capacity and overall tree vigor (Figure 1).

**Figure 2:**
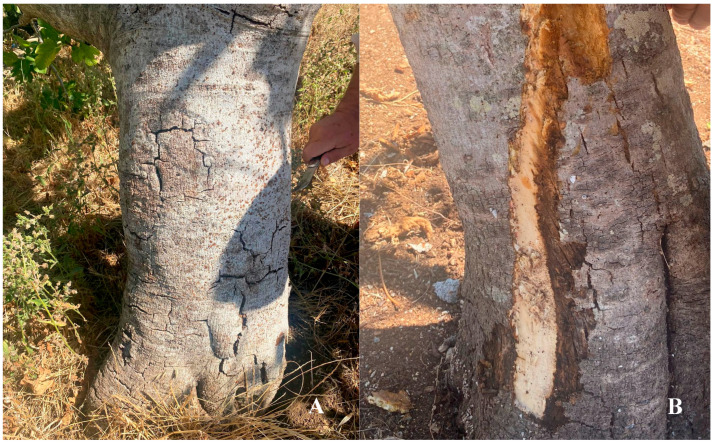
Symptoms of *C. ficicola* infection observed on the basal portion of the trunk of fig trees. (**A**) External appearance of the bark, showing longitudinal cracks and dark, sunken lesions coalescing around the stem above the soil line. (**B**) Internal symptoms revealed after bark removal, showing extensive brown discoloration of the vascular tissue, particularly the xylem, and signs of dark exudate, indicative of pathogen colonization and vascular disruption.

At the crown root region, just above the soil line, the bark frequently exhibited dark brown to nearly black necrotic lesions. These lesions expanded and often coalesced, occasionally girdling the trunk and disrupting vascular continuity. Longitudinal cracks developed in advanced stages, sometimes accompanied by the exudation of dark brown sap. Removal of the outer bark consistently revealed diffuse brown discoloration of the vascular tissues, particularly the xylem, which extended upward from the crown (Figure 2A,B). These alterations are indicative of pathogen colonization and vascular disruption. In severe cases, such extensive vascular impairment leads to progressive decline and ultimately tree mortality if unmanaged.

Fungal isolates obtained from diseased tissues were grouped according to morphological criteria into three main clusters—*Fusarium* spp., Botryosphaeriaceae, and Ceratocystidaceae—while taxa not assignable to these groups were collectively listed as “Other fungi.”

Isolates of the cultures grouped as belonging to the *Fusarium* cluster typically exhibit fast-growing, woolly to cottony aerial mycelia that range in color from white to cream, often developing shades of light violet, blue–green, or pale brown with age. The colony reverse is usually dark bluish green to brownish, occasionally with a diffusible pigment that may color the medium. These cultures produced macroconidia, typically falcate (sickle-shaped) to straight, with three to five septa, and exhibited a blunt to slightly curved apical cell and a distinct foot-shaped basal cell. The size of macroconidia varied depending on the isolates, but in general macroconidia measured approximately 42.5 ± 6.25 × 5.25 ± 0.63 µm, with three to five septa (sometimes up to seven). Microconidia were generally oval, ellipsoidal to cylindrical, and aseptate or occasionally one-septate (Figure S1). They were produced abundantly in false heads on long monophialides arising from aerial hyphae. Their dimensions were 8.5 ± 1.75 × 3.0 ± 0.5 µm. Based on these morphological characteristics, the cultures were preliminarily grouped as belonging to the *Fusarium solani* species complex (FSSC) [27,29].

Cultures belonging to the Botryosphaeriaceae displayed septate, branched mycelia that ranged in color from white to gray, typically darkening with age. Within this family, two growth patterns were evident. The first group grew rapidly, forming fluffy, cotton-like colonies that began as white and progressively turned gray to dark olive, often producing a diffusible pigmented exudate. The second group showed slower growth, with colonies shifting gradually from white–gray to dark brown or black. Both types produced submerged or aerial mycelia of variable architecture. After 10–15 days of incubation on pine needle agar, pycnidia formed, dark in color and variable in size and shape, most commonly globose to subglobose with a distinct ostiolar opening.

Within these cultures, one subset of isolates produced thin-walled, hyaline conidia, ellipsoid to fusiform and aseptate, and measuring on average 24.0 ± 3.0 × 6.0 ± 0.5 µm. As they matured, conidia became light brown and one-septate [47,48]. Based on these traits, the isolates were provisionally identified as *Neofusicoccum* spp. Another group produced slightly larger conidia (27.0 ± 2.89 × 8.0 ± 1.45 µm), initially hyaline, fusiform to ellipsoidal, and aseptate, but later pigmented and septate, resembling *Diplodia*. These were tentatively assigned to *Botryosphaeria* spp. [47,48].

Colonies of Ceratocystidaceae fungi grew rapidly, reaching diameters of 40–60 mm within 5–7 days, and typically exhibited dark olive to black pigmentation with a fluffy to appressed texture. Sexual structures, specifically long-necked, globose to subglobose perithecia, began to develop after approximately 7–10 days of incubation. Mature perithecia exuded cream- to amber-colored cirri composed of ascospores, which were ellipsoidal to fusiform and measured 6.0 ± 0.5 × 3.5 ± 0.25 µm. Endoconidia were produced within phialides and typically appeared within 3–5 days. These conidia were hyaline, cylindrical, and measured approximately 6.0 ± 1.0 × 2.5 ± 0.25 µm. Chlamydospores, which were thick-walled and globose, measured approximately 10.0 ± 1.0 µm in diameter and developed intercalarily or terminally after 10–14 days, particularly in aging cultures. While these morphological features supported the preliminary identification of the isolates as belonging to *Ceratocystis* spp., definitive species-level identification was accomplished through multilocus phylogenetic analysis based on the ITS, EF-1α, β-tubulin, and RPB2 gene regions [43,49].

### 2.2. Frequency and Relative Abundance of Isolated Morphological Groups

Results of the mycological analyses conducted on wood fragments and adult beetles collected from six sites (A–F) in the provinces of Lecce and Brindisi, Apulia, are reported in Table 1. Isolates were assigned to four groups: the *Fusarium solani* species complex (FSSC), *Ceratocystis* spp., Botryosphaeriaceae (including *Neofusicoccum* spp.), and “Others.” FSSC dominated wood samples, with recovery rates up to 90%, but was rarely associated with beetles, where detections occasionally reached 50%. *Ceratocystis* spp. were less frequent in wood (≈7.5%), yet showed comparable recovery in beetles (≤50%), suggesting insect-mediated transmission. Botryosphaeriaceae were widespread in wood (32%–90%), but nearly absent in beetles (12%). Notably, multiple fungal taxa co-occurred in 67% of samples, pointing to frequent mixed infections and complex pathogen interactions within host tissues.

### 2.3. Phylogenetic Analyses and Species Identification

**Figure 3:**
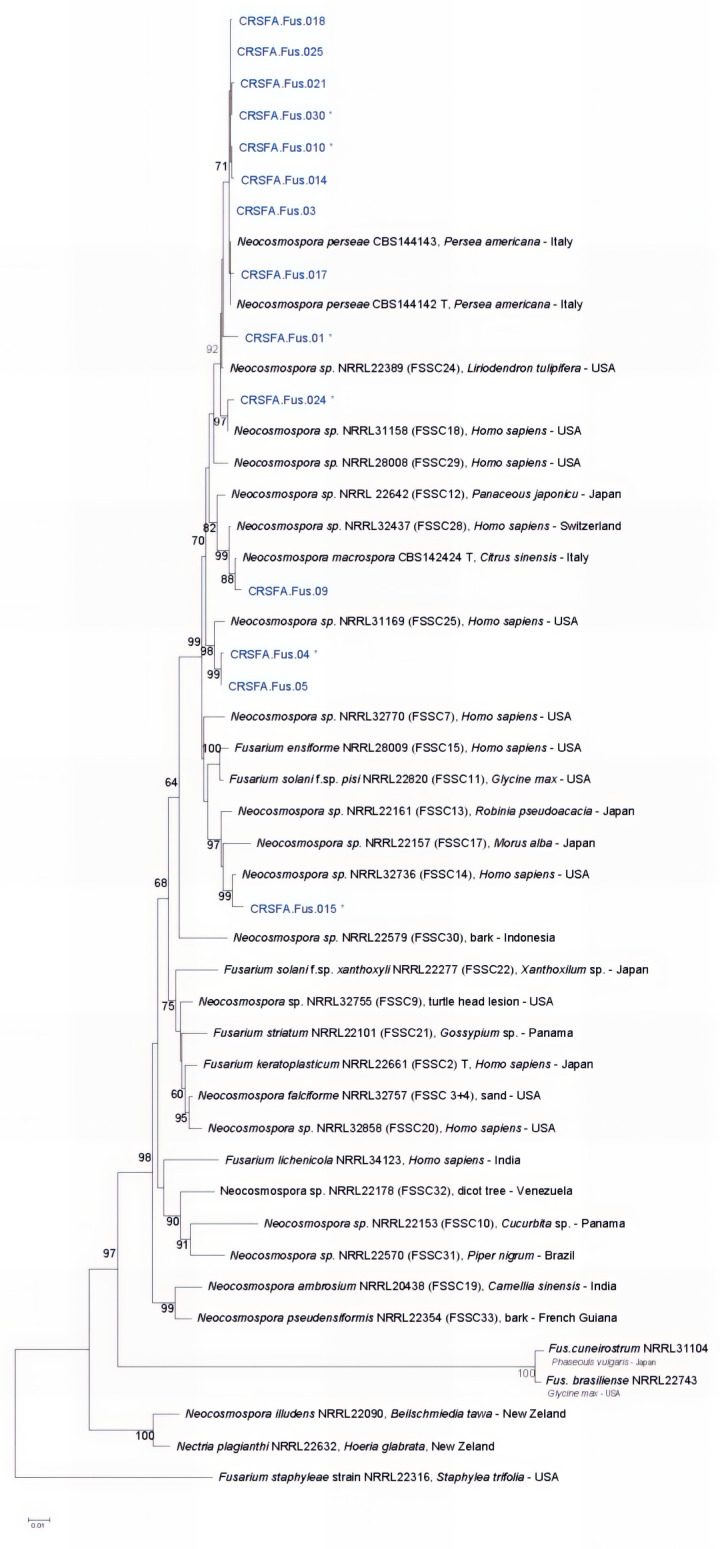
Maximum-likelihood phylogenetic tree of the *Fusarium solani* species complex (FSSC) based on the Tamura–Nei model. Bootstrap values (%) indicate branch support, and branch lengths represent substitutions per site. The analysis included 48 sequences with 2185 aligned positions. Black characters = reference strains; blue characters = field isolates. T = Ex-type strains; * isolates tested for pathogenicity.

Pairwise sequence alignments using BLASTn 2.15.0 searches in GenBank confirmed that the fungal isolates belonged to the genera *Neofusicoccum*, *Botryosphaeria*, *Lasiodiplodia*, *Neocosmospora*, and *Ceratocystis*. Representative isolates of Botryosphaeriaceae, the *Fusarium solani* species complex, and Ceratocystidaceae were subjected to multilocus sequence analyses for more comprehensive taxonomic resolution. For the fusarioid fungal isolates, multilocus phylogenetic analyses were conducted based on four gene regions: EF-1α, ITS, LSU, and RPB2. The final combined alignment included 48, comprising 14 field strains, 33 reference sequences, and one outgroup taxon (*Fusarium staphyleae*), resulting in an alignment of 2185 characters, of which 1369 were conserved and 800 were variable and phylogenetically informative. The phylogenetic analysis of the concatenated sequences yielded a tree (Figure 3) rooted with the outgroup that resolved three well-defined clades, each supported by high bootstrap values. Specifically, eight field strains (CRSFA.Fus.017, CRSFA.Fus.03, CRSFA.Fus.010, CRSFA.Fus.014, CRSFA.Fus.021, CRSFA.Fus.030, CRSFA.Fus.018, and CRSFA.Fus.025) clustered with *N. perseae* (Figure S1).

Additionally, CRSFA.Fus.01 grouped with *Neocosmospora* sp. FSSC 24; CRSFA.Fus.024 with *Neocosmospora* sp. FSSC 18; CRSFA.Fus.015 with *Neocosmospora* sp. FSSC 14; and CRSFA.Fus.04 and CRSFA.Fus.05 with *Neocosmospora* sp. FSSC 25. Finally, CRSFA.Fus.09 grouped with *N. macrospora*.

**Figure 4:**
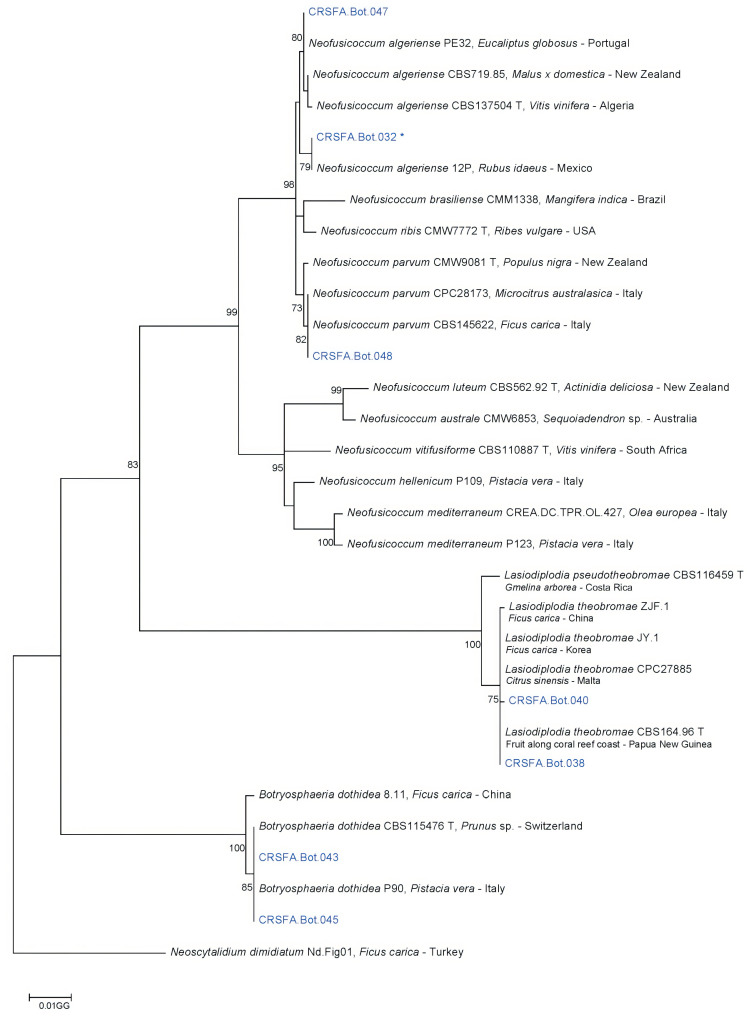
Maximum-likelihood phylogenetic tree of Botryosphaeriaceae isolates based on the Tamura–Nei model. Bootstrap values (%) indicate branch support, and branch lengths represent substitutions per site. The analysis included 31 sequences with 1062 aligned positions. Black characters = reference strains; blue characters = field isolates. T = Ex-type strains; * isolates tested for pathogenicity.

The Botryosphaeriaceae isolates were phylogenetically analyzed using three informative gene regions: ITS, EF-1α, and TUB2. The final sequence alignment of the combined dataset included 31 isolates, comprising seven field strains, 23 reference sequences, and one strain of *Neoscytalidium dimidiatum* used as an outgroup. The alignment consisted of 1062 characters, of which 225 were variable and phylogenetically informative. The phylogenetic analysis of the concatenated sequences from the three loci yielded a tree (Figure 4) in which two field strains, CRSFA.Bot.047 and CRSFA.Bot.032, clustered with reference strains of *N. algeriense* (Figure S2). Strain CRSFA.Bot.048 grouped with *N. parvum* reference strains. Strains CRSFA.Bot.040 and CRSFA.Bot.038 clustered with *L. theobromae* reference strains, while the remaining two field strains, CRSFA.Bot.043 and CRSFA.Bot.045, grouped with reference strains of *B. dothidea*.

**Figure 5:**
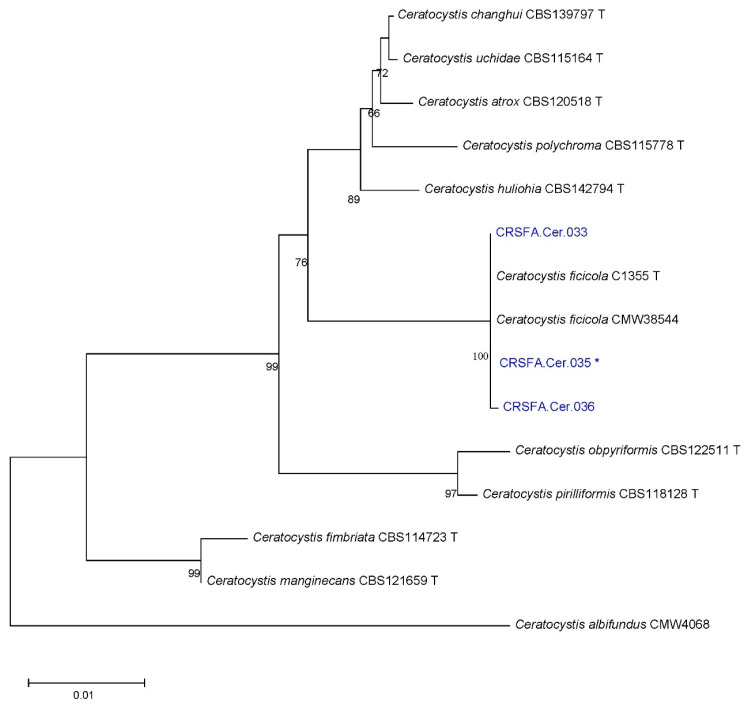
Maximum-likelihood phylogenetic tree of *Ceratocystis* spp. isolates based on the Tamura–Nei model (highest log likelihood = −3389.78). Bootstrap values (%) indicate branch support, and branch lengths represent substitutions per site. The analysis included 15 sequences with 1544 aligned positions. Black characters = reference strains; blue characters = field isolates. T = Ex-type strains; * isolates tested for pathogenicity.

Finally, for Ceratocystidaceae, multilocus phylogenetic analyses were performed based on three gene regions: TEF-1α, ITS, and RPB2. The final sequence alignment of the combined dataset included 15 isolates, comprising three field strains, 12 reference sequences, and one strain of *C. albifundus* used as an outgroup. The alignment consisted of 1950-character sites, of which 324 were variable and phylogenetically informative. The phylogenetic analysis of the concatenated sequences from the three loci resulted in a tree (Figure 5) rooted with the outgroup. All field strains of Ceratocystidaceae clustered with reference strains of *C. ficicola* (Figure S3).

The results of both mycological and molecular analyses indicated that among *Fusarium* species, *N. perseae* (Figure S1) was the most frequently recovered, with an isolation rate of 58% from wood fragments and 38.5% from beetles. Other *Fusarium* species were recovered at frequencies ranging from 5.8% to 38.5%.

Among Botryosphaeriaceae, *B. dothidea* and *L. theobromae* were the most frequently isolated from wood, with recovery rates of 61.4% and 31.8%, respectively. *L. theobromae* was also recovered from beetles at a frequency of 100%. *N. parvum* and *N. algeriense* (Figure S2) were isolated from wood fragments at frequencies of 5% and 2.3%, respectively.

All field strains of Ceratocystidaceae were identified as *C. ficicola* (Figure S3).

### 2.4. Pathogenicity Tests Results 

**Figure 6:**
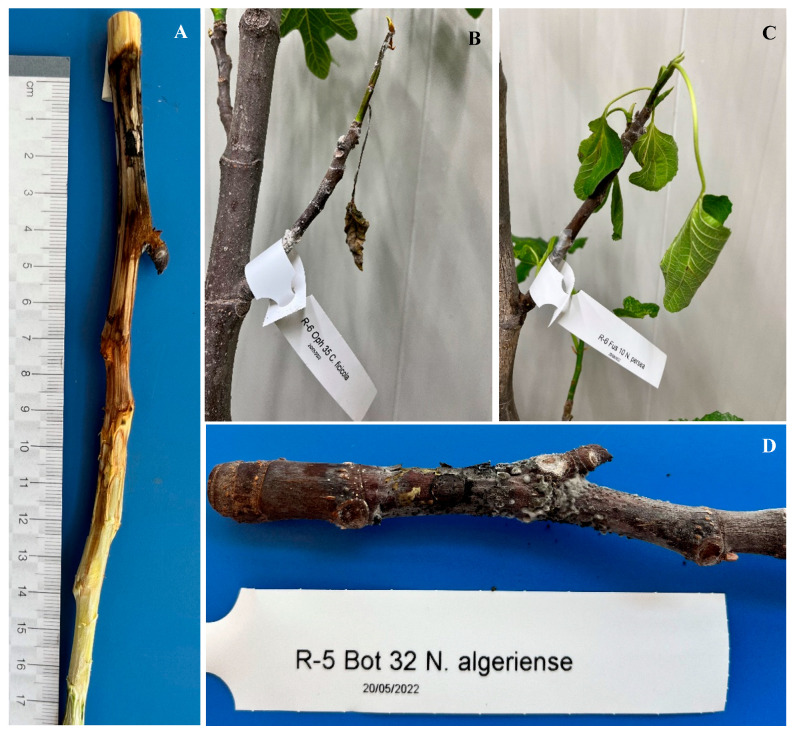
Pathogenicity tests of *C. ficicola* (**A**,**B**), *N. perseae* (**C**), and *N. algeriense* (**D**) on potted *Ficus carica* plants. Inoculations were performed by inserting a mycelial plug into a 6 mm wound on young twigs. After one month, both external symptoms (chlorosis, wilting, dieback) and internal symptoms (vascular discoloration beneath the bark) were evident on inoculated twigs, confirming the pathogenicity of the tested isolates.

Pathogenicity tests revealed that not all fusarioid field strains were pathogenic to fig, with the exception of *N. perseae* isolates CRSFA.Fus.010 and CRSFA.Fus.030 (Figure 6C). In all six replicates, initial symptoms appeared one month after inoculation. After 39 days, symptomatic twigs were sectioned, and colonization was quantified by measuring wood discoloration (length h and diameter d) using the formula V = π × (d/2)^2^ × h. *N. perseae* isolates CRSFA.Fus.010 and CRSFA.Fus.030 showed colonization of twig surfaces ranging from 56.3% to 86.5%. The mean colonized surface area for *N. perseae* isolates was 71.7% ± 0.1%. The pathogenicity of *N. algeriense* (CRSFA.Bot.032) was also confirmed, with initial symptoms developing in all six replicates one month after inoculation (see Figure 6D). After 39 days, the average colonized surface area was 61.5% ± 0.1%. Similarly, *C. ficicola* (CRSFA.Cer.035) induced visible symptoms one month post-inoculation, and by day 39, the colonized surface area reached 64.0% ± 0.1% (see Figure 6A,B). Data on pathogenicity test are summarized in Table 2.

Fungi employed in the pathogenicity tests were successfully re-isolated from all inoculated twigs, thereby confirming their consistent recovery and association with the induced symptoms. On the whole, the percentage of colonized surface relative to total twig surface revealed clear differences among pathogens: *N. perseae* isolates reached the highest levels of colonization (mean 71.7% ± 3.1%), followed by *C. ficicola* (64.0% ± 0.1%) and *N. algeriense* (61.5% ± 0.1%), thus confirming their differential virulence on fig.

### 2.5. Identification of Insects

Observation of morphological characteristics of adult specimens under a stereomicroscope led to the identification of the bark beetle *C. dilutus* (Eichhoff). A distinctive trait useful for rapid identification is the presence of a spine on the mesofemur of the male, a feature exclusive to this species [40,50] and observed in all males examined. Species identification was corroborated by sequencing of the cytochrome C oxidase subunit I (COX1) gene (GenBank accession no. PQ333917). This record confirms the occurrence of the bark beetle *C. dilutus* in the Apulia region, verified through molecular characterization based on cytochrome C oxidase subunit I (COX1) gene sequences.

## 3. Discussion

Symptoms of wilt and decline in fig (*Ficus carica*) were first recorded in the Salento area of Apulia in September 2021 [41] and consistently monitored through 2025. Affected plants displayed a progressive disease syndrome, beginning with foliar chlorosis and wilting, followed by premature defoliation, twig dieback, and death of lateral branches. Advanced stages were marked by bark cracking, extensive cankers, and pronounced wood discoloration in trunk and branch cross sections. The fungal communities isolated from fig wood and associated beetles comprised a diverse assemblage with varying ecological roles and pathogenic potential. Based on morphological and molecular analysis, fungal isolates recovered from symptomatic figs were identified as *Neocosmospora* spp., with *N. perseae* being the most frequent, followed by *C. ficicola, N. algeriense, N. parvum,* and *B. dothidea*.

Morphological characteristics combined with a robust four-locus phylogenetic analysis (ITS, EF-1α, LSU, and RPB2) as per Guarnaccia et al. [29] identified *N. perseae* as the most frequently isolated species within the *Neocosmospora* genus. Species of this genus are known to affect a broad range of hosts, including plants, animals, and humans [25,51]. Several *Neocosmospora* species are commonly associated with crown and/or root rot in infected plants. Symptoms on aerial plant parts may include cankers, wilting, stunting, chlorosis, and lesions on stems and/or leaves, often resulting in significant economic losses [29,52].

*Fusarium* and *Neocosmospora* species are widespread in Italian nurseries [53,54,55,56,57], where they represent a significant constraint on the production of ornamental plants. In addition, several *Neocosmospora* species have been reported in Italy as pathogens of fruit crops, including citrus, fig, and avocado [26,29,30].

Recently, *N. parceramosa* was identified on raspberry in northern Italy, where it was associated with cane blight disease and shown to produce symptoms in artificially inoculated raspberry plants [31].

Between 2010 and 2014, surveys carried out in ornamental plant production areas of eastern Sicily led to the isolation of *N. solani* from crown and root rot symptoms in fig plants [30].

Within the *Neocosmospora* genus, two additional species, *N. caricae* and *N. metavorans*, have recently been reported as pathogens of fig in Iran. These species are responsible for stem and trunk canker as well as fig decline, affecting commercial orchards in the region [32].

The species *N. perseae* identified in this study was previously described in Italy as a novel species responsible for trunk cankers on avocado in Sicily [29]. In 2020, branch canker symptoms were observed in Crete, Greece, and the causal agent was similarly identified as *N. perseae*. In the present study, pathogenicity tests confirmed the role of *N. perseae* as a pathogen of fig in Italy, thereby fulfilling Koch’s postulates. Other *Fusarium solani* species complex (FSSC) isolates included in the pathogenicity assays did not induce disease symptoms, suggesting that they may act as endophytes in fig or potentially function as symbionts in insect development and reproduction. This possibility aligns with previously reported symbioses between *Euwallacea* beetles and *Fusarium* species, such as the *F. kuroshium*–*E. interjectus* association documented in several countries [58].

In the present study, species of the family Botryosphaeriaceae were identified through multilocus phylogenetic analysis using ITS, EF-1α, and TUB sequences. *Botryosphaeria dothidea* was the most frequently isolated species from wood tissues, followed by *L. theobromae* and *N. parvum*.

Both *B. dothidea* and *L. theobromae* have previously been reported in Italy on various host plants. *B. dothidea* has been found on sycamore (*Platanus occidentalis*, L.), red oak (*Quercus rubra* L.), and English oak (*Quercus robur* L.) [59], as well as on grapevine [13], red eucalyptus in Sardinia (*Eucaliptus camaldulensis* Dehnh) [60], and pistachio (*Pistacia vera* L.) [61]. *N. parvum* is a well-documented, aggressive pathogen affecting several economically important crops, including citrus, avocado, and blueberry (*Vaccinium caesariense* Rydb.) [62]. Similarly, *L. theobromae* has been reported on grapevine and avocado [63,64].

In 2022, *L. theobromae* and *B. dothidea* were detected for the first time in Italy on mango, where they were associated with woody canker and shoot blight. These species were also found on litchi in Sicily, along with other Botryosphaeriaceae species already reported in the region, such as *N. parvum*.

Several Botryosphaeriaceae species identified in the current investigation, including *B. dothidea*, *L. theobromae*, and *N. parvum*, have previously been reported as pathogens of fig. In particular, *N. parvum* was associated with twig blight, subcortical discoloration, and apical dieback in *Ficus carica* plants sampled in June 2018 from a nursery in Catania Province, Sicily [14]. *B. dothidea* and *N. parvum* have also been reported on *Ficus microcarpa* in Italy, causing severe branch cankers and dieback [65]. Additionally, *L. theobromae* has been reported on fig in China and South Korea [17,18], while *B. dothidea* was also reported on fig in China [15].

Among the Botryosphaeriaceae species investigated in this study, *N. algeriense* was recovered from fig for the first time in Italy. Previously, it had been reported on grapevine in Algeria, where it causes grapevine trunk disease [66], on *Eucalyptus globulus* in Portugal [67], and on raspberry in Mexico as the causal agent of dieback [68]. In the present study, pathogenicity tests confirmed its pathogenic role on fig.

From symptomatic fig tissues, a species belonging to the family Ceratocystidaceae was isolated and identified as *C. ficicola* through morphological analysis and multilocus phylogenetic analysis based on three informative loci (ITS, EF-1α, and RPB2). Although less frequently isolated, *C. ficicola* is of particular concern. Recently, it was reported in Greece, where it was first detected in orchards in the Attica region and subsequently identified on Euboea Island in 2019 [44], causing wilt epidemics. While typically linked to wounds or insect galleries [69], its soilborne biology indicates that infection via the root system cannot be excluded. Its recovery from beetles at two sites—representing up to 50% of isolates—further points to insect-mediated spread.

First observed in Japan during the 1970s [70] and formally described only in 2011 [43], this species has caused severe outbreaks, leading some growers to abandon fig orchards, with comparable levels of tree mortality also reported in Greece. Since February 2022, *C. ficicola* has been included in the EPPO Alert List due to its potential to cause severe tree mortality and the difficulty of its eradication [71]. Overall, all the recent findings on this pathogen highlight a dual epidemiological pathway combining soil/root transmission with vector-assisted dispersal and reinforce the urgency of stringent phytosanitary measures.

Therefore, further research is required to elucidate the biology and epidemiology of this species, as well as to develop a rapid and reliable method for its detection.

Wood samples collected from symptomatic trees revealed the presence of numerous bark beetle galleries and detached, necrotic bark. Within the small galleries under the decorticated bark, various life stages of the insect—including larvae, pupae, adults, and occasionally eggs—were observed. One bark beetle species was identified and characterized both morphologically and molecularly as *C. dilutus* Eichhoff. *C. dilutus* was consistently found at all sampling sites where bark beetle exit holes were present. *Cryphalus dilutus* is a thermophilic species of Asian origin, first described in the Indian subcontinent. It has since been reported in India, Bangladesh, China, the United Arab Emirates, Pakistan, Oman, Mexico, and Israel [40,50]. The species was first detected in Europe (Malta) in 1991 [72], likely introduced accidentally through the importation of ornamental *Ficus microcarpa* plants, which also serve as a host for the beetle [73]. In Malta, approximately 50–70% of fig trees have been affected by this insect [50,74]. In 2014, *C. dilutus* was reported in Italy (Sicily) [8], followed by detections in France in 2017 [75] and Tunisia in 2018 [76]. A total of 52 *C. dilutus* specimens collected during this study were also subjected to mycological analysis. Several fungal species isolated from symptomatic fig tissues were also recovered from the beetles. Specifically, *L. theobromae*, *N. perseae*, and *C. ficicola* were isolated from *C. dilutus* specimens.

At present, no confirmed vector relationship exists between *N. perseae* and any insect. However, given that the *Neocosmospora* genus includes other economically significant tree-pathogenic mutualists of shot-hole borers (e.g., *Euwallacea* spp.) [28], further investigation into a possible association with *C. dilutus* is warranted.

Moreover, considering that other species of Ophiostomatoid fungi are known to be transmitted by insect vectors—such as *Ophiostoma ulmi* and *O. novo-ulmi*, which are vectored by elm bark beetles (*Scolytus scolytus* and *S. multistriatus*) [36,37,38], and *C. fimbriata*, which along with *L. theobromae* causes mango sudden death disease in Pakistan and is vectored by the bark beetle *C. dilutus* (*Hypocryphalus mangifera*) [39]—further studies are needed to clarify the potential role of *C. dilutus* in the transmission of fungal pathogens.

The potential of an arthropod to act as a vector can be investigated in future studies by applying the four criteria outlined by Leach [77], which are conceptually compatible with Koch’s postulates [78]. Testing these postulates involves conducting a bioassay in which the putative arthropod vector is allowed to colonize healthy test plants, followed by the assessment of fungal transmission by the bark beetle.

The consistent recovery of bark beetles, along with various fungal species from different genera, from the same symptomatic fig tissues suggests that fig decline may result from a mixed infection involving both insect vectors and pathogenic fungi.

In addition to the newly tested fungi, our isolations frequently yielded *B. dothidea* and *L. theobromae*, two species already demonstrated as active pathogens of fig in different geographic contexts [14,15,17,46,65]. Therefore, we did not repeat pathogenicity tests for these taxa, but their high isolation frequency in Apulia confirms their involvement in fig decline and further supports the view of a disease with mixed fungal etiology. Diseases arising from mixed infections, in which multiple pathogens interact within the same host, pose challenges for diagnosis and management because of the overlapping symptoms and intricate biological interactions involved.

A key feature of diseases of mixed etiology lies in the temporal sequence of pathogen infections within the host, which strongly influences symptom expression and disease progression. The order in which each pathogen colonizes the host, along with their respective trophic levels, may significantly influence the interactions that develop during disease progression. Synergistic interactions among different fungal pathogens have been well documented in the literature.

Although several pathogenic fungi were isolated in this study, the evidence supports the involvement of bark beetle species as potential vectors. This hypothesis is particularly important for guiding future research aimed at clarifying the mechanisms of fungal transmission.

## 4. Materials and Methods

### 4.1. Field Sampling and Isolation

Plant material was collected from different fig orchards (Table 1). Specifically, samples were collected from Salice Salentino (Site A), Copertino (Site B), Guagnano (Site C); Ostuni (Site D), Squinzano (Site E), and Ceglie Messapica (Site F). The sampled fig trees belonged to locally cultivated varieties, predominantly *Dottato Bianco*. Except for Site F1, where the trees were relatively young (5–6 years old), the remaining trees were mature (30–50 years old) and grown in non-specialized orchards. All samples of diseased fig trees were collected between April and October during each year of observation (2021–2025) across the different locations listed in Table 1.

Wood fragments (5 × 5 mm) from symptomatic fig tissues were excised from the lesion margins, surface-sterilized by immersion in 70% ethanol for 30 s, followed by treatment with 1% sodium hypochlorite for 60 s, and finally rinsed twice with sterile water. The tissue fragments were dried on sterile filter paper and transferred onto potato dextrose agar (PDA; composed of 200 g/L boiled potato extract, 20 g/L D-(+)-glucose, and 20 g/L agar), supplemented with 0.5 g/L streptomycin sulphate (Sigma-Aldrich S.R.L., Steinheim, Germany). The plates were incubated at 25 ± 1 °C until fungal colonies developed. Colonies originating from wood fragments were then transferred onto fresh PDA plates to establish pure cultures. To obtain a monoconidial isolate from each representative fungal culture, a small fragment of a sporulating colony was transferred into a sterile tube containing a 0.01% Tween 20 sterile solution. The suspension was gently agitated to release the conidia from the mycelium. To ensure adequate separation of individual spores, the conidial suspension was appropriately diluted and evenly spread onto the surface of a water agar plate using a sterile spreader. The plates were then incubated at 25 ± 1 °C in the dark or light for 12–24 h or until germination of the conidia occurred. Under a stereomicroscope, a single germinated conidium was identified and transferred aseptically using a sterile needle to fresh potato dextrose agar (PDA). The monoconidial isolates were then incubated at 25 ± 1 °C until a visible colony developed. This culture, derived from a single spore, represents a genetically uniform isolate and was used for further morphological, molecular, or pathogenicity analyses.

At all sites except B and D, the trees showed signs of bark beetle infestation. Consequently, branches and wood containing insect galleries were specifically sampled. The collected specimens were stored in 70% ethanol at −20 °C. Additionally, 52 adult bark beetle specimens were processed for fungal isolation and production of monoconidial isolates using the same methodology as described above.

Overall, a total of 24 representative fungal isolates (Table 3) were selected for macro- and micromorphological characterization based on their traits and subsequent phylogenetic analysis.

### 4.2. Morphological Characterization and Preliminary Identification of the Fungal Isolates

The morphological characterization of monosporic cultures was carried out through the observation of colony morphology and the microscopic examination of conidia and other reproductive structures (e.g., pycnidia). Pure cultures were grouped into distinct morphological categories based on both macroscopic and microscopic features.

In particular, the identification of *Ophiostomatoid*-like cultures was based on colony growth patterns, pigmentation, and the development of reproductive structures on different media, including malt extract agar (MEA) and potato dextrose agar (PDA). Cultures were incubated at 25 ± 1 °C in darkness for 7 to 14 days to promote sporulation [33,43].

The identification of *Fusarium*-like cultures was based on colony morphology and conidial features under standardized conditions. Cultures were grown on PDA, synthetic nutrient-poor agar (SNA), and carnation leaf agar (CLA) and incubated at 25 ± 1 °C under a 12 h light/12 h dark photoperiod for 7 to 10 days. Microscopic analysis focused on the shape, size, and septation of macroconidia and microconidia, as well as the presence of chlamydospores [26].

For the identification of Botryosphaeriaceae species, particularly *Neofusicoccum* and *Botryosphaeria* spp., cultures were established on PDA and water agar (WA) amended with sterilized pine needles. Incubation was conducted at 25 ± 1 °C under a 12 h photoperiod for 7 to 14 days to induce pycnidia formation and conidiation [9,79].

Conidial measurements were performed under a compound light microscope at 200× and 400× magnification. For each fungal isolate, at least 100 conidia were randomly selected from 10 independent optical fields to ensure representative sampling. Conidia were mounted in sterile distilled water and examined under both bright-field and phase-contrast illumination. Conidial length and width were recorded using an integrated digital imaging system equipped with calibrated measurement software following standard protocols for fungal morphological characterization [80].

### 4.3. DNA Extraction and PCR Amplification and Sequencing

#### 4.3.1. DNA Extraction

Each fungal isolate was inoculated onto cellophane membranes placed over PDA using five mycelial plugs collected from 2- to 3-day-old cultures. The plates were then incubated in the dark at 21 ± 1 °C for a minimum of two days. Genomic DNA was extracted and purified from the mycelia grown on the cellophane membranes following the protocol described by Habib et al. [81]. The resulting DNA pellets were washed with 70% ethanol, air-dried, and subsequently dissolved in sterile distilled water. DNA concentrations were determined using a NanoDrop 2000 spectrophotometer (Thermo Fisher Scientific Inc., Wilmington, DE, USA), and samples were stored at −20 °C until further use.

#### 4.3.2. Amplification and Sequencing

For each fungal group, three to four informative gene regions were amplified using optimized PCR conditions, as described in Table 4. In the case of *Fusarium* isolates, fragments from four nuclear loci were targeted: the internal transcribed spacer region of the rDNA (ITS), translation elongation factor 1 alpha (TEF-1α), the large subunit of the rDNA (LSU), and the second-largest subunit of RNA polymerase II (RPB2). PCR amplifications were carried out in 25 µL reaction volumes, each containing 1× Taq PCR Ready Master Mix (Qiagen), 0.4 µM of each primer (0.2 µM for ITS), and 1 µL of genomic DNA (2 µL for RPB2) at a concentration of 25 ng/µL. Reactions targeting the ITS and RPB2 regions additionally included 3 mM MgCl_2_.

For members of the Ceratocystidaceae family, fragments of three nuclear loci (ITS, TEF-1α, and RPB2) were PCR-amplified using the same protocol as described previously (Table 4). For Botryosphaeriaceae, fragments of three nuclear loci (ITS, TEF-1α, and β-tubulin [TUB2]) were amplified. PCR conditions for TEF-1α followed the same protocol as outlined above. For TUB2 amplification, PCR reactions were performed in 25 µL volumes, each containing 1× Taq PCR Ready Master Mix (Qiagen, Hilden, Germany), 0.4 µM of each primer, 1.5 mM MgCl_2_, and 1 µL of genomic DNA extract (25 ng/µL). The PCR products were stained with SYBR™ Safe DNA Gel Stain (Invitrogen by Thermo Fisher Scientific) and visualized after electrophoretic separation on a 1.0% agarose gel in 1× TAE buffer. Visualization was performed under UV light, and bands were compared to a 100 bp DNA ladder (Thermo Fisher Scientific). All PCR products were sequenced bidirectionally by an external service (Genewiz, www.genewiz.com).

### 4.4. Phylogenetic Analysis and Molecular Identification

The sequences of the isolates were edited and proofread using Chromas 2.1.10.1 software (www.chromas.it). Preliminary species identification was conducted via BLAST analysis against the GenBank nucleotide database. Sequence alignment and manual adjustments were carried out using MEGA 11 software [88]. To investigate the phylogenetic relationships among *Fusarium solani* species complex, Botryosphaeriaceae, and Ceratocystidaceae species, the sequences obtained in this study were aligned with reference sequences using the Clustal W algorithm. For the *Fusarium solani* species complex, 33 reference strains were included, with *Fusarium staphyleae* NRRL 22316 used as the outgroup (Table S1). For Botryosphaeriaceae, 24 reference strains were used, with *Neoscytalidium dimidiatum* Nd Fig01 serving as the outgroup (Table S2). In the case of Ceratocystidaceae, 11 reference sequences were analyzed, with *C. albifundus* strain CMW4068 designated the outgroup (Table S3).

All reference sequences were retrieved from the National Center for Biotechnology Information (NCBI) and incorporated into the phylogenetic analyses.

Phylogenetic relationships were inferred using three to four informative gene regions and analyzed with the maximum-likelihood method based on the Tamura–Nei model [48], implemented in MEGA software version 11. Bootstrap analysis was performed to assess the reliability of internal nodes using an heuristic search with 1000 replicates. Alignment gaps were excluded from the analysis.

### 4.5. Pathogenicity Tests

Pathogenicity tests were carried out on young twigs (1–2 years old) of healthy *Ficus carica* cv. Dottato plants grown under controlled greenhouse conditions (19–25 °C, ~70% relative humidity, natural daylight). The fig plantlets used for the pathogenicity test were grown in a well-drained sandy-loam soil typical of Apulia derived from the local agricultural soil, to which one-third peat was added to improve organic matter and water retention. The soil mixture was sterilized prior to use to eliminate potential interfering microorganisms. Each selected fungal isolate was inoculated onto six replicate plants with three to five twigs per plant, providing a robust and reproducible design for the consistent evaluation of symptom development across tissues. The test was performed following the method described by Bolboli et al. [32].

Briefly, a 6 mm wound was made using a sterilized cork-borer, and a 6 mm-diameter mycelial plug, taken from the margin of a 5-day-old PDA culture, was inserted into the wound. The inoculation site was then sealed with Parafilm to prevent desiccation and contamination. Non-colonized PDA plugs were used as negative controls.

After 30 days, symptoms began to develop, and symptomatic twigs were returned to the laboratory for further analysis. Disease symptoms were assessed, and fungal pathogens were re-isolated on PDA to confirm their identity and fulfill Koch’s postulates.

All inoculated twigs were sectioned longitudinally, and the bark was carefully removed above and below the inoculation points. The length (h) and diameter (d) of wood discoloration were measured (Figure 6A), and the volume of colonized tissue was calculated using the formula V = π × (d/2)^2^ × h.

In addition, the percentage of the colonized surface relative to the total twig surface was determined using the formula:

% Colonized Surface = 100 × d/D,

where d = diameter of the colonized area and D = external diameter of the twig.

To confirm Koch’s postulates, re-isolations were carried out from symptomatic tissues collected from the inoculated plants. Small wood fragments taken from the margins of discolored tissues at varying distances from the inoculation point were surface-sterilized as described before and aseptically transferred to PDA amended with 0.5 g/L streptomycin sulfate. Emerging fungal colonies were examined for cultural and morphological traits and compared with those of the original inoculated isolates. Representative re-isolates were further identified by sequencing the ITS region and additional loci (e.g., EF-1α, β-tubulin, or RPB2, depending on the genus). The re-isolated strains consistently matched the inoculated ones, thereby completing Koch’s postulates.

### 4.6. Identification of Scolytid Species

From the plant samples, approximately one hundred individuals at different biological stages—larvae, pupae, numerous adults, and in some cases even eggs—were recovered. Species identification was performed by examining morphological characteristics of adult specimens following the guidelines described by Johnson et al. [40,50]. All specimens were examined under a Nikon SMZ745T stereomicroscope at 50× magnification.

Morphological identification was subsequently confirmed through molecular analysis. DNA was extracted from selected insects following the protocol outlined in EPPO Standard PM 7/24 (4) [89]. For molecular identification, the mitochondrial cytochrome C oxidase subunit I (COI) gene was amplified using the primer pair LCO1490–HCO2198 [90].

PCR amplifications were carried out in 25 µL reaction volumes, each containing 1× Taq PCR Ready Master Mix (Qiagen, Hilden, Germany), 0.2 µM of each primer, and 1 µL of genomic DNA extract (25 ng/µL). Cycling conditions followed those reported in EPPO Standard PM 7/129 (2) [91]. All amplifications were performed using a T100 thermal cycler (Bio-Rad, Hercules, CA, USA), and sequencing was conducted by GENEWIZ Europe (Leipzig, Germany).

The highest-quality sequences obtained were compared with reference sequences deposited in the Barcode of Life Data System (BOLD) database (www.boldsystems.org).

## Figures and Tables

**Table 1:** Percentage and origin of fungal colonies isolated from fig wood and adult beetles (*Cryphalus dilutus*) samples.

Site	Locality	Province	Sample	Wood Fragments (#)	Adult Beetles (#)	Percentage of Fungal Colonies Recovered							
						*Fusarium* spp.		*Ceratocystis* spp.		Botryosphaeriaceae		Others	
						Wood	Beetles	Wood	Beetles	Wood	Beetles	Wood	Beetles
A	Salice Salentino	Lecce	A-1	30	9	46.67	66.67	6.00	0.00	3.33	0.00	13.33	0.00
			A-2	10	3	30.00	33.33	2.00	0.00	0.00	0.00	10.00	0.00
B	Copertino	Lecce	B-1	40	-	12.50	-	0.00	-	77.50	-	0.00	-
C	Guagnano	Lecce	C-1	30	30	0.00	0.00	0.00	0.00	93.33	12.00	0.00	0.00
D	Ostuni	Brindisi	D-1	25	-	0.00	-	0.00	-	92.00	-	0.00	-
E	Squinzano	Lecce	E-1	30	0	90.00	0.00	0.00	0.00	3.33	0.00	0.00	0.00
			E-2	10	5	90.00	30.00	2.00	0.00	0.00	0.00	0.00	0.00
			E-3	5	5	0.00	20.00	5.00	20.00	0.00	0.00	0.00	0.00
			E-4	20	0	55.00	0.00	1.00	0.00	20.00	0.00	15.00	0.00
F	Ceglie Messapica	Brindisi	F-1	40	0	17.50	0.00	7.50	0.00	32.50	0.00	0.00	0.00
			F-2	30	2	36.67	50.00	6.67	50.00	36.67	0.00	0.00	0.00
			F-3	40	0	25.00	0.00	5.00	0.00	22.50	0.00	0.00	0.00

**Table 2:** Pathogenicity of representative fungal isolates on *Ficus carica* cv. Dottato twigs under greenhouse conditions. Values represent the mean percentage of colonized twig surface (±standard error, SE) with ranges across six replicates. Disease symptoms were consistently observed in all plants inoculated with *Neocosmospora perseae*, *Neofusicoccum algeriense*, and *Ceratocystis ficicola* isolates, while other fusarioid strains were non-pathogenic. Figure references indicate representative symptom development.

Fungal Species (Isolate Code)	Colonized Twig Surface (%) ± SE	Notes/Figure Reference
*Neocosmospora perseae* (CRSFA.Fus.010)	74.2 ± 3.2 (range: 65.5–81.5)	Symptoms in all replicates; see Figure 6C
*Neocosmospora perseae* (CRSFA.Fus.030)	69.2 ± 3.0 (range 60.9–77.2)	Symptoms in all replicates; see Figure 6C
*Neofusicoccum algeriense* (CRSFA.Bot.032)	61.5 ± 2.1 (range 54.0–68.0)	Confirmed pathogenicity; see Figure 6D
*Ceratocystis ficicola* (CRSFA.Cer.035)	64.0 ± 1.8% (range 58–70%)	Symptoms in all replicates; see Figure 6A,B
Other fusarioid strains (various)	Non-pathogenic	No symptoms observed

**Table 3:** Collection details and GenBank accession numbers of isolates subjected to multi-gene phylogeny.

Species	Culture Code	Host	Locality	Province	Site	Sample	GenBank Number				
							ITS	TEF	RPB2	TUB	LSU
*Botryosphaeria dothidea*	CRSFA.Bot.043	*F. carica*	Copertino	Lecce	B	B-1	OQ644314	OQ716469	-	OQ716476	-
	CRSFA.Bot.045	*F. carica*	Ostuni	Brindisi	D	D-1	OQ646770	OQ716470	-	OQ716477	-
*Ceratocystis ficicola*	CRSFA.Cer.033	*F. carica*	Salice Salentino	Lecce	A	A-1	OQ329983	OQ352265	OQ352268	-	-
	CRSFA.Cer.035	*F. carica*	Squinzano	Lecce	E	E-4	OQ335969	OQ352266	OQ352267	-	-
	CRSFA.Cer.036	*Cryphalus dilutus*	Squinzano	Lecce	E	E-3	OQ939559	OQ944935	OQ944934	-	-
*Lasiodiplodia theobromae*	CRSFA.Bot.040	*C. dilutus*	Guagnano	Lecce	C	C-1	OQ644250	OQ716472	-	OQ716474	-
	CRSFA.Bot.038	*F. carica*	Guagnano	Lecce	C	C-1	OQ646769	OQ657193	-	OQ716475	-
*Neofusicoccum algeriense*	CRSFA.Bot.047	*F. carica*	Squinzano	Lecce	E	E-1	OQ642317	OQ657192	-	OQ657191	-
	CRSFA.Bot.032	*F. carica*	Salice Salentino	Lecce	A	A-1	OQ652029	OQ716471	-	OQ716478	-
*N. macrospora*	CRSFA.Fus.09	*F. carica*	Salice Salentino	Lecce	A	A-1	OQ780916	OQ934042	OQ944931		OQ818831
*N. parvum*	CRSFA.Bot.048	*F. carica*	Squinzano	Lecce	E	E-4	OQ642323	OQ674076	-	OQ716473	-
*N. perseae*	CRSFA.Fus.03	*F. carica*	Copertino	Lecce	B	B-1	OQ708344	OR354697	OQ984265	-	OQ818623
	CRSFA.Fus.010	*F. carica*	Salice Salentino	Lecce	A	A-1	OQ785882	OR294029	OR003942	-	OQ845689
	CRSFA.Fus.014	*F. carica*	Squinzano	Lecce	E	E-2	OQ785883	OR344502	OR159909	-	OQ842738
	CRSFA.Fus.017	*C. dilutus*	Squinzano	Lecce	E	E-2	OQ818688	OR344503	OR168709	-	OQ845690
	CRSFA.Fus.018	*C. dilutus*	Squinzano	Lecce	E	E-3	OQ818687	OR354698	OR188777	-	OQ845693
	CRSFA.Fus.021	*F. carica*	Squinzano	Lecce	E	E-4	OQ818685	OR344504	OR188778	-	OQ845692
	CRSFA.Fus.025	*F. carica*	Squinzano	Lecce	E	E-1	OQ818690	OR365292	OR188779	-	OQ845687
	CRSFA.Fus.030	*F. carica*	Squinzano	Lecce	E	E-1	OQ818689	OR365293	OR188780	-	OQ845691
*Neocosmospora* sp. (FSSC 24)	CRSFA.Fus.01	*F. carica*	Copertino	Lecce	B	B-1	OQ708490	OQ885478	OQ944929	-	OQ818180
*Neocosmospora* sp. (FSSC 25)	CRSFA.Fus.04	*C. dilutus*	Salice Salentino	Lecce	A	A-1	OQ708492	OQ934040	OQ984264	-	OQ818664
	CRSFA.Fus.05	*C. dilutus*	Salice Salentino	Lecce	A	A-1	OQ750696	OQ934041	OQ944930	-	OQ818686
*Neocosmospora* sp. (FSSC 14)	CRSFA.Fus.015	*F. carica*	Squinzano	Lecce	E	E-2	OQ790083	OQ934043	OQ944932	-	OQ842766
*Neocosmospora* sp. (FSSC 18)	CRSFA.Fus.024	*F. carica*	Squinzano	Lecce	E	E-3	OQ816805	OQ934044	OQ944933	-	OQ845688

Not available. CRSFA = Centro di Ricerca, Sperimentazione e Formazione in Agricoltura “Basile Caramia.”

**Table 4:** PCR conditions for primers used in this study.

Amplified Gene	Primers Pairs	Reference	Optimized PCR Protocols	Fungal Pathogens
ITS	ITS4 ITS5	[82]	95°: 5 min, (95° C: 1 min, 58° C: 1 min, 72° C: 1 min) × 25 cycles, 72 °C: 7 min	*Fusarium* spp. Botryosphaeriaceae *Ceratocystis* spp.
LSU	LR0R LR5	[83]	95 °C: 5 min, (95 °C: 1 min, 55 °C: 1 min, 72 °C: 1 min) × 25 cycles, 72 °C: 7 min	*Fusarium* spp.
TEF-1α	Tef1 Tef2	[84]	95 °C: 5 min, (95 °C: 1 min, 57 °C: 75 s, 72 °C: 1 min) × 30 cycles, 72 °C: 10 min	*Fusarium* spp. *Ceratocystis* spp.
	ef1-986R ef1-728F	[85]	95 °C: 5 min, (95 °C: 1 min, 57 °C: 75 s, 72 °C: 1 min) × 30 cycles, 72 °C: 10 min	Botryosphaeriaceae
*rpb2*	fRPB2-5F fRPB2-7cR	[86]	95 °C: 5 min, (95 °C: 1 min, 55 °C: 1 min, 72 °C: 1 min) × 25 cycles, 72 °C: 7 min	*Fusarium* spp. *Ceratocystis* spp.
*β*-tubulin	Bt2a Bt2b	[87]	94 °C: 3 min, (94 °C: 40 s, 58 °C: 1 min, 72 °C: 2 min) × 35 cycles, 72 °C: 10 min	Botryosphaeriaceae

## Data Availability

The original contributions presented in this study are included in the article/Supplementary Material. Further inquiries can be directed to the corresponding author.

## Supplementary Materials

The following supporting information can be downloaded at https://www.mdpi.com/article/10.3390/plants14182865/s1, Table S1: Collection details and GenBank accession numbers of *Fusarium* spp. isolates included in the multi-gene phylogenetic analysis; Table S2: Origin and GenBank accession numbers of *Botryosphaeriaceae* reference strains included in the multi-gene phylogenetic analysis; Table S3: Collection details and GenBank accession numbers of *Ceratocystis* isolates included in the multi-gene phylogenetic analysis; Figure S1: Morphological characteristics of *Neocosmospora perseae* CRSFA.Fus.017 isolated from symptomatic *Ficus carica* branch; Figure S2: Morphological features of *Neofusicoccum algeriense* from symptomatic fig plant; Figure S3: Morphological characteristics of *Ceratocystis ficicola* CRSFA_Cer_035.

## References

[1] Kislev M.E., Hartmann A., Bar-Yosef O. (2006). Early domesticated fig in the Jordan Valley. Science.

[2] Flaishman M., Rodov V., Stover E. (2008). The fig: Botany, horticulture, and breeding. Hortic. Rev..

[3] Condit R. (1995). Research in large, long-term tropical forest plots. Trends Ecol. Evol..

[4] Çaliskan O., Polat A.A. (2011). Phytochemical and Antioxidant properties of selected fig (*Ficus carica* L.) accessions from the Eastern Mediterranean Region of Turkey. Sci. Hortic..

[5] Mazzeo A., Magarelli A., Ferrara G. (2024). The fig (*Ficus carica* L.): Varietal evolution from Asia to Puglia region, southeastern Italy. CABI Agric. Biosci..

[6] García M.M., Denno B.D., Miller D.R., Miller G.L., Ben-Dov Y., Hardy N.B. (2016). ScaleNet: A literature-based model of scale insect biology and systematics. Database.

[7] Farina P., Mazza G., Benvenuti C., Cutino I., Giannotti P., Conti B., Bedini S., Gargani E. (2021). Biological notes and distribution in Southern Europe of *Aclees taiwanensis* Kono, 1933 (Coleoptera: Curculionidae): A new pest of the fig tree. Insects.

[8] Faccoli M., Campo G., Perrotta G., Rassati D. (2016). Two newly introduced tropical bark and ambrosia beetles (Coleoptera: Curculionidae, Scolytinae) damaging figs (*Ficus carica*) in southern Italy. Zootaxa.

[9] Slippers B., Wingfield M.J. (2007). Botryosphaeriaceae as endophytes and latent pathogens of woody plants: Diversity, ecology and impact. Fungal Biol. Rev..

[10] von Arx J.A. (1987). Plant Pathogenic Fungi.

[11] Carlucci A., Raimondo M.L., Cibelli F., Phillips J.L., Lops F. (2013). *Pleurostomophora richardsiae*, *Neofusicoccum parvum* and *Phaeoacremonium aleophilum* associated with a decline of olives in southern Italy. Phytopathol. Mediterr..

[12] Mondello V., Lo Piccolo S., Conigliaro G., Alfonzo A., Torta L., Burruano S. (2013). First report of *Neofusiccoccum vitifusiforme* and presence of other Botryosphaeriaceae species associated with *Botryosphaeria* dieback of grapevine in Sicily (Italy). Phytopathol. Mediterr..

[13] Carlucci A., Cibelli F., Lops F., Raimondo M.L. (2015). Characterization of Botryosphaeriaceae species as causal agents of trunk diseases on grapevines. Plant Dis..

[14] Aiello D., Gusella G., Fiorenza A., Guarnaccia V., Polizzi G. (2020). Identification of *Neofusicoccum parvum* causing canker and twig blight on *Ficus carica* in Italy. Phytopathol. Mediterr..

[15] Wang X., Zhang X., Li M., Ji X., Feng C., Wang F. (2020). First Report of *Ficus carica* bot rot caused by *Botryosphaeria dothidea* in China. Plant Dis..

[16] Çeliker N.M., Michailides T.J. (2012). First report of *Lasiodiplodia theobromae* causing canker and shoot blight of fig in Turkey. New Dis. Rep..

[17] Chen Y., Wei H., Du G., Zhu L., Song Q., Hu Y., Wang E., Wang M., Fan X. (2018). First Report of *Lasiodiplodia theobromae* causing stem canker on common fig (*Ficus carica*) in Zhejiang Province of China. Plant Dis..

[18] Seo Y., Back C.G., Park M.J., Park J.H. (2019). First Report of *Lasiodiplodia theobromae* causing canker and dieback of common fig in Korea. Plant Dis..

[19] Guarnaccia V., Vitale A., Cirvilleri G., Aiello D., Susca A., Epifani F., Perrone G., Polizzi G. (2016). Characterisation and pathogenicity of fungal species associated with branch cankers and stem-end rot of avocado in Italy. Eur. J. Plant Pathol..

[20] Ismail A.M., Cirvilleri G., Lombard L., Crous P.W., Groenewald J.Z., Polizzi G. (2013). Characterisation of *Neofusicoccum* species causing mango dieback in Italy. Plant Pathol. J..

[21] Elshafie E.A., Ba-Omar T. (2001). First report of Albizia lebbeck dieback caused by *Scytalidium dimidiatum* in Oman. Mycopathologia.

[22] Ray J.D., Burgess T., Lanoiselet V.M. (2010). First record of *Neoscytalidium dimidiatum* and *N. novaehollandiae* on *Mangifera indica* and *N. dimidiatum* on *Ficus carica* in Australia. Australas. Plant Dis. Notes.

[23] Gusella G., Fiore G., Vitale A., Felts D.G., Michailides T.J. (2023). New findings on the effects of different factors involved in fig limb dieback caused by *Neoscytalidium dimidiatum* in California. Eur. J. Plant Pathol..

[24] Michielse C.B., Rep M. (2009). Pathogen profile update: *Fusarium oxysporum*. Mol. Plant Pathol..

[25] O’Donnell K., Sutton D.A., Fothergill A., McCarthy D., Rinaldi M.G., Brandt M.E., Zhang N., Geiser D.M. (2008). Molecular phylogenetic diversity, multilocus haplotype nomenclature, and in vitro antifungal resistance within the *Fusarium solani* species complex. Clin. Microbiol. J..

[26] Sandoval-Denis M., Guarnaccia V., Polizzi G., Crous P.W. (2018). Symptomatic Citrus trees reveal a new pathogenic lineage in *Fusarium* and two new *Neocosmospora* species. Persoonia-Mol. Phylogeny Evol. Fungi.

[27] Sandoval-Denis M., Lombard L., Crous P.W. (2019). Back to the roots: A reappraisal of *Neocosmospora*. Persoonia-Mol. Phylogeny Evol. Fungi.

[28] Freeman S., Sharon M., Maymon M., Mendel Z., Protasov A., Aoki T., Eskalen A., O’Donnell K. (2013). *Fusarium euwallaceae* sp. nov.—A symbiotic fungus of *Euwallacea* sp., an invasive ambrosia beetle in Israel and California. Mycologia.

[29] Guarnaccia V., Sandoval-Denis M., Aiello D., Polizzi G., Crous P.W. (2018). *Neocosmospora perseae* sp. nov., causing trunk cankers on avocado in Italy. Fungal Syst. Evol..

[30] Guarnaccia V., Aiello D., Polizzi G., Crous P.W., Sandoval-Denis M. (2019). Soilborne diseases caused by *Fusarium* and *Neocosmospora* spp. on ornamental plants in Italy. Phytopathol. Mediterr..

[31] Guarnaccia V., Martino A., Brondino L., Gullino M.L. (2022). *Paraconiothyrium fuckelii*, *Diaporthe eres* and *Neocosmospora parceramosa* causing cane blight of red raspberry in Northern Italy. Plant Pathol..

[32] Bolboli Z., Mostowfizadeh-Ghalamfarsa R., Sandoval-Denis M., Jafari M., Crous P.W. (2022). *Neocosmospora caricae* sp. *nov.* and *N. metavorans*, two new stem and trunk canker pathogens on *Ficus carica* in Iran. Mycol. Prog..

[33] De Beer Z.W., Wingfield M.J., Seifert K.A., De Beer Z.W., Wingfield M.J. (2013). Emerging lineages in the Ophiostomatales. The Ophiostomatoid Fungi: Expanding Frontiers.

[34] Kajii C., Morita T., Jikumaru S., Kajimura H., Yamaoka Y., Kuroda K. (2013). Xylem dysfunction in *Ficus carica* infected with wilt fungus *Ceratocystis ficicola* and the role of the vector beetle *Euwallacea interjectus*. IAWA J..

[35] Moller W.J., Devay J.E. (1968). Carrot as a species-selective isolation medium for *Ceratocystis fimbriata*. Phytopathology.

[36] Favaro A., Battisti A. (1993). Observation on elm bark beetle, *Scolytus pymaeus* (Fabricius) (*Coleoptera*, *Scolytidae*) as possible vectors of the fungus *Ophiostoma ulmi* (Schwarz) Nannfeldt. Redia.

[37] Battisti A., Favaro A., Faccoli M., Masuitti L. (1994). Suscettibilità di ceppi di *Ulmus* spp. all’attacco primario di *Scolytus* sp. Pl. (*Coleoptera, Scolytidae*). Innovazioni e Prospettive Nella Difesa Fitosanitaria.

[38] Faccoli M., Battisti A., Geogoire J.C., Liebhold A.M., Stephen F.M., Day K.R., Salom S.M. (1997). Observation on the transmission of *Ophiostoma ulmi* by the smaller Elm Bark Beetles (*Scolytus* spp.). Integrating Cultural Tactics into the Management of Bark Beetles and Reforestation Pest.

[39] Masood A., Saeed S. (2012). Bark beetle, *Hypocryphalus mangiferae* stebbing (Coleoptera: Curculionidae: Scolytinae) is a vector of mango sudden death disease in Pakistan. Pak. J. Bot.

[40] Johnson A.J., Li Y., Mandelshtam M.Y., Park S., Lin C.S., Gao L., Hulcr J. (2020). East Asian *Cryphalus Erichson* (*Curculionidae, Scolytinae*): New species, new synonymy and redescriptions of species. ZooKeys.

[41] Habib W., Cavalieri V., Carlucci M., Dongiovanni C., Nigro F. (2022). A new disease complex threatening fig (*Ficus carica* L.) in Southern Italy. 16th Congress of the Mediterranean Phytopathological Union, 4–8 April 2022, Limassol, Cyprus. Phytopathol. Mediterr..

[42] Gugliuzzo A., Gusella G., Leonardi G.R., Costanzo M.B., Ricupero M., Rassati D., Biondi A., Polizzi G. (2023). From a cause of rapid fig tree dieback to a new threat to mango production: The invasive bark beetle *Cryphalus dilutus* Eichhoff (Coleoptera: *Curculionidae, Scolytinae*) and its associated fungi found on mango trees in Europe. EPPO Bull..

[43] Kajitani Y., Masuya H. (2011). *Ceratocystis ficicola* sp. nov., a causal fungus of fig canker in Japan. Mycoscience.

[44] Tsopelas P., Soulioti N., Wingfield M.J., Barnes I., Marincowitz S., Tjamos E.C., Paplomatas E.J. (2021). *Ceratocystis ficicola* causing a serious disease of *Ficus carica* in Greece. Phytopathol. Mediterr..

[45] Habib W., Carlucci M., Manco L., Altamura G., Delle Donne A.G., Nigro F. (2023). First report of *Ceratocystis ficicola* causing canker and wilt disease on common fig (*Ficus carica*) in Italy. Plant Dis..

[46] Crous P.W., Akulov A., Balashov S., Boers J., Braun U., Castillo J., Delgado M.A., Denman S., Erhard A., Gusella G. (2023). New and interesting Fungi. 6. Fungal Syst. Evol..

[47] Hattori Y., Ando Y., Nakashima C. (2021). Taxonomical re-examination of the genus *Neofusicoccum* in Japan. Mycoscience.

[48] Phillips A.J.L., Alves A., Abdollahzadeh J., Slippers B., Wingfield M., Groenewald J., Crous P.W. (2013). The Botryosphaeriaceae: Genera and species known from culture. Stud. Mycol..

[49] De Beer Z.W., Duong T.A., Barnes I., Wingfield B.D., Wingfield M.J. (2014). Redefining *Ceratocystis* and allied genera. Stud. Mycol..

[50] Johnson A.J., Knížek M., Atkinson T.H., Jordal B.H., Ploetz R.C., Hulcr J. (2017). Resolution of a global mango and fig pest identity crisis. Insect Syst. Divers..

[51] Lombard L., Van der Merwe N.A., Groenewald J.Z., Crous P.W. (2015). Generic concepts in Nectriaceae. Stud. Mycol..

[52] Coleman J.J. (2016). The *Fusarium solani* species complex: Ubiquitous pathogens of agricultural importance. Mol. Plant. Pathol..

[53] Polizzi G., Vitale A. (2003). First report of *Fusarium* blight on majesty palm caused by *Fusarium proliferatum* in Italy. Plant Dis..

[54] Polizzi G., Aiello D., Guarnaccia V., Vitale A., Perrone G., Stea G. (2010). First report of Fusarium wilt of paper flower (*Bougainvillea glabra*) caused by *Fusarium oxysporum* in Italy. Plant Dis..

[55] Polizzi G., Aiello D., Guarnaccia V., Vitale A., Perrone G., Epifani F. (2010). First report of Fusarium wilt on *Eremophila* spp. caused by *Fusarium oxysporum* in Italy. Plant Dis..

[56] Polizzi G., Aiello D., Guarnaccia V., Vitale A., Perrone G., Stea G. (2011). First report of Fusarium wilt on *Philotheca myoporoides* caused by *Fusarium oxysporum* in Italy. Plant Dis..

[57] Bertoldo C., Gilardi G., Spadaro D., Gullino M.L., Garibaldi A. (2015). Genetic diversity and virulence of Italian strains of *Fusarium oxysporum* isolated from *Eustoma grandiflorum*. Eur. J. Plant Pathol..

[58] Jiang Z.R., Masuya H., Kajimura H. (2021). Novel symbiotic association between *Euwallacea ambrosia* beetle and *Fusarium* fungus on fig trees in Japan. Front. Microbiol..

[59] Turco E., Marianelli L., Vizzuso C., Ragazzi A., Gini R., Selleri B., Tucci R. (2006). First report of *Botryosphaeria dothidea* on sycamore, red oak, and english oak in North western Italy. Plant Dis..

[60] Deidda A., Buffa F., Linaldeddu B.T., Pinna C., Scanu B., Deiana V., Satta A., Franceschini A., Floris I. (2016). Emerging pests and diseases threaten *Eucalyptus camaldulensis* plantations in Sardinia, Italy. Forest.

[61] Gusella G., Lawrence D.P., Aiello D., Luo Y., Polizzi G., Michailides T. (2021). Etiology of *Botryosphaeria panicle* and shoot blight of pistachio (*Pistacia vera*) caused by Botryosphaeriaceae in Italy. Plant Dis..

[62] Guarnaccia V., Martino I., Tabone G., Brondino L., Gullino M.L. (2020). Fungal pathogens associated with stem blight and dieback of blueberry in northern Italy. Phytopathol. Mediterr..

[63] Burruano S., Mondello V., Conigliaro G., Alfonzo A., Spagnolo A., Mugnai L. (2008). Grapevine decline in Italy caused by *Lasiodiplodia theobromae*. Phytopathol. Mediterr..

[64] Garibaldi A., Bertetti D., Amatulli M.T., Cardinale J., Gullino M.L. (2012). First report of postharvest fruit rot in avocado (*Persea americana*) caused by *Lasiodiplodia theobromae* in Italy. Plant Dis..

[65] Fiorenza A., Aiello D., Costanzo M.B., Gusella G., Polizzi G. (2022). A New Disease for Europe of *Ficus microcarpa* Caused by Botryosphaeriaceae Species. Plants.

[66] Berraf-Tebbal A., Guereiro M.A., PHilliPS A.J., Von Arx J.A. (2014). Phylogeny of *Neofusicoccum* species associated with grapevine trunk diseases in Algeria, with description of *Neofusicoccum algeriense* sp. nov. Phytopath. Mediterr..

[67] Barradas C., Phillips A.J., Correia A., Diogo E., Bragança H., Alves A. (2016). Diversity and potential impact of Botryosphaeriaceae species associated with *Eucalyptus globulus* plantations in Portugal. Eur. J. Plant Pathol..

[68] Serret-López R.E., Tlapal-Bolaños B., Leyva-Mir S.G., Correia K.C., Camacho-Tapia M., Méndez-Jaimes F., Tovar-Pedraza J.M. (2017). First report of *Neofusicoccum algeriense* causing dieback of red raspberry in Mexico. Plant Dis..

[69] Masuya H., Yamaoka Y., Kaneko S., Yamaura Y. (2009). Ophiostomatoid fungi isolated from Japanese red pine and their relationships with bark beetles. Mycoscience.

[70] Kato K., Hirota K., Miyagawa T. (1982). A new disease, Ceratocystis canker of fig caused by Ceratocystis fimbriata Ellis et Halsted. Plant Prot..

[71] (2022). EPPO Alert List—*Ceratocystis ficicola*. https://www.eppo.int/ACTIVITIES/plant_quarantine/alert_list_fungi/ceratocystis_ficicola.

[72] Mifsud D., Knizek M. (2009). The Bark Beetles (*Coleoptera: Scolytidae*) of the Maltese Islands (Central Mediterranean). Bull. Ent. Soc. Malta.

[73] Mifsud D., Annushka Falzon A., Malumphy C., de Lillo E., Vovlas N., Porcelli F. (2012). On some arthropods associated with *Ficus* species (Moraceae) in the Maltese Islands. Bull. Ent. Soc. Malta.

[74] Bonanno G. (2016). Alien species: To remove or not to remove? That is the question. Environ. Sci. Policy.

[75] Barnouin T., Soldati F., Roques A., Faccoli M., Kirkendall L.R., Mouttet R., Daubree J.B., Noblecourt T. (2020). Bark beetles and pinhole borers recently or newly introduced to France (Coleoptera: Curculionidae, Scolytinae and Platypodinae). Zootaxa.

[76] Gaaliche B., Ben Abdelaali N., Mouttet R., Ben Halima-Kamel M., Hajlaoui M.R. (2018). First report of Hypocryphalus scabricollis (Eichhoff, 1878), in Tunisia, an emerging pest on fig trees (*Ficus carica* L.). Bull. OEPP/EPPO Bull..

[77] Leach J.G. (1940). Insect Transmission of Plant Diseases.

[78] Whetzel H.H. (1918). An Outline of the History of Phytopathology.

[79] Slippers B., Crous P.W., Jami F., Groenewald J.Z., Wingfield M.J. (2017). Diversity in the Botryosphaeriales: Looking back, looking forward. Fungal Biol..

[80] Crous P.W., Wingfield M.J., Burgess T.I., Hardy G.E.S.J. (2016). Fungal Planet description sheets: 400–468. Persoonia.

[81] Habib W., Masiello M., El Ghorayeb R., Gerges E., Susca A., Meca G., Quiles M.J., Logrieco F.A., Moretti A. (2021). Mycotoxin profile and phylogeny of pathogenic *Alternaria* species isolated from symptomatic tomato plants in Lebanon. Toxins.

[82] White T.J., Bruns T.D., Lee S.B., Taylor J.W. (1990). Amplification and Direct Sequencing of Fungal Ribosomal RNA Genes for Phylogenetics. PCR Protoc. A Guide Methods Appl..

[83] Vilgalys R., Hester M. (1990). Rapid genetic identification and mapping of enzymatically amplified ribosomal DNA from several *Cryptococcus species*. J. Bacteriol..

[84] O’Donnell K., Kistler H.C., Cigelnik E., Ploetz R.C. (1998). Multiple evolutionary origins of the fungus causing Panama disease of banana: Concordant evidence from nuclear and mitochondrial gene genealogies. Proc. Natl. Acad. Sci. USA.

[85] Carbone I., Kohn L.M.A. (1999). Method for designing primer sets for speciation studies in filamentous ascomycetes. Mycologia.

[86] Liu Y.J., Whelen S., Hall B.D. (1999). Phylogenetic relationships among ascomycetes: Evidence from an RNA polymerse II subunit. Mol. Biol. Evol..

[87] Glass N.L., Donaldson G.C. (1995). Development of primer sets designed for use with the PCR to amplify conserved genes from filamentous ascomycetes. Appl. Environ. Microbiol..

[88] Tamura K., Stecher G., Kumar S. (2021). MEGA11: Molecular Evolutionary Genetics Analysis Version 11. Mol. Biol. Evol..

[89] EPPO (2019). PM 7/24 (4) *Xylella fastidiosa*. EPPO Bull..

[90] Folmer O., Black M., Hoeh W., Lutz R., Vrijenhoek R. (1994). DNA primers for amplification of mitochondrial cytochrome c oxidase subunit I from diverse metazoan invertebrates. Mol. Mar. Biol. Biotechnol..

[91] EPPO (2021). PM 7/129 (2) DNA barcoding as an identification tool for a number of regulated pests. EPPO Bull..

