# Supplementary material for: Unveiling a Disease Complex Threatening Fig (Ficus carica L.) Cultivation in Southern Italy
Source: Plants (Basel). 2025 Sep 15;14(18):2865. doi: 10.3390/plants14182865 (PMC12473328; doi:10.3390/plants14182865)
Supplement: Supplementary file 1 [file plants-14-02865-s001.zip › plants-3859317-supplementary.pdf]

plants

MDPI

Article

Unveiling a Disease Complex Threatening Fig (*Ficus carica* L.) Cultivation in Southern Italy

Wassim Habib [1], Mariangela Carlucci[2], Vincenzo Cavalieri [1,3], Cecilia Carbotti[2], and Franco Nigro [1,2]

Table S1. Collection details and GenBank accession numbers of *Fusarium* spp. isolates included in the multi-gene phylogenetic analysis.

Species	Clade number	Strain	Locality	Host	GenBank accession numbers			
					ITS	TEF	LSU	RPB2
Fusarium brasiliense		NRRL 22743	USA	*Glycine max*	EF408512	EF408407	FJ919502	EU329525
F. cuneirostrum		NRRL 31104	Japan	*Phaseolus vulgaris*	FJ919509	EF408413	FJ919509	EU329558
F. ensiforme	FSSC 15	NRRL 28009	USA	*Homo sapiens*	DQ094351	DQ246869	DQ236393	EF470136
F. keratoplasticum	FSSC 2 T *	NRRL 22661	Japan	*Homo sapiens*	DQ094331	DQ246846	DQ236373	EU329524
F. lichenicola	FSSC 16	NRRL 34123	Indian	*Homo sapiens*	DQ094645	DQ247192	DQ236687	EU329635
F. solani f.sp. pisi	FSSC 11	NRRL 22820	USA	*Glycine max*	DQ094310	AF178355	DQ236352	EU329532
F. solani f.sp. xanthoxyli	FSSC 22	NRRL 22277	Japan	*Xanthoxylum* sp.	AF178401	AF178336	AF178370	FJ240380
F. staphyleae		NRRL 22316	USA	*Staphylea trifolia*	MH582406	MH582426	AF178392	EU329502
F. striatum	FSSC 21	NRRL 22101	Panama	*Gossypium sp.*	AF178398	AF178333	AF178367	EU329490
Neocosmospora ambrosium	FSSC 19	NRRL 20438	India	*Camellia sinensis*	AF178397	AF178332	DQ236357	JX171584
N. falciforme	FSCC 3+4	NRRL 32757	USA	Sand	DQ094536	DQ247075	DQ236578	EU329614
N. illudens		NRRL 22090	New Zeland	*Beilschmiedia tawa*	AF178393	AF178326	AF178362	JX171601
N. macrospora	T	CBS 142424	Italy	*Citrus sinensis*	LT746266	LT746218	LT746281	LT746331
N. perseae	T	CBS 144142	Italy	*Persea americana*	LT991940	LT991902	LT991947	LT991909
N. perseae		CBS 144143	Italy	*Persea americana*	LT991941	LT991903	LT991948	LT991910
N.ra plagianthi		NRRL 22632	New Zeland	*Hoeria glabrata*	AF178354	AF178354	AF178386	JX171614
N. pseudensiformis	FSSC 33	NRRL 22354	French Guiana	Bark	AF178402	AF178338	DQ236358	EU329504
Neocosmospora sp.	FSSC 7	NRRL 32770	USA	*Homo sapiens*	DQ094544	DQ247083	DQ236586	EU329615
Neocosmospora sp.	FSSC 9	NRRL 32755	USA	Turtle head lesion	DQ094534	DQ247073	DQ236576	EU329613

Neocosmospora sp.	FSSC 10	NRRL 22153	Panama	*Cucurbita L.* sp.	DQ094302	AF178346	DQ236344	EU329492
Neocosmospora sp.	FSSC 13	NRRL 22161	Japan	*Robinia pseudoacacia*	DQ094329	DQ246844	DQ236371	EU329522
Neocosmospora sp.	FSSC 12	NRRL 22642	Japan	*Penaceous japonicu*	DQ094329	DQ246844	DQ236371	EU329522
Neocosmospora sp.	FSSC 14	NRRL 32736	USA	*Homo sapiens*	DQ094517	DQ247056	DQ236559	EU329605
Neocosmospora sp.	FSSC 17	NRRL 22157	Japan	*Morus alba*	DQ094306	AF178359	DQ236348	EU329493
Neocosmospora sp.	FSSC 18	NRRL 31158	USA	*Homo sapiens* Hn	DQ094389	DQ246916	DQ236431	EU329559
Neocosmospora sp.	FSSC 20	NRRL 32858	USA	*Homo sapiens*	DQ094617	DQ247163	DQ236659	EU329630
Neocosmospora sp.	FSSC 24	NRRL 22389	USA	*Liriodendron tulipifera*	AF178404	AF178340	DQ236356	EU329506
Neocosmospora sp.	FSSC 25	NRRL 31169	USA	*Homo sapiens*	DQ094396	DQ246923	DQ236438	KR673999
Neocosmospora sp.	FSSC 28	NRRL 32437	Switzerland	*Homo sapiens*	DQ094446	DQ246979	DQ236488	EU329581
Neocosmospora sp.	FSSC 29	NRRL 28008	USA	*Homo sapiens*	DQ094350	DQ246868	DQ236392	EF470135
Neocosmospora sp.	FSSC 30	NRRL 22579	Indonesia	Bark	AF178415	AF178352	AF178384	EU329515
Neocosmospora sp.	FSSC 31	NRRL 22570	Brazil	*Piper nigrum L.* sp.	AF178422	AF178360	AF178391	EU329513
Neocosmospora sp.	FSSC 32	NRRL 22178	Venezuela	Dicot tree	AF178399	AF178334	AF178368	EU329498

*: T= Ex-type strains.

Table S2. Origin and GenBank accession numbers of *Botryosphaeriaceae* reference strains included in the multi-gene phylogenetic analysis.

Species	Strain	Locality	Host plant	GenBank accession numbers		
				**ITS**	**TEF**	**TUB**
Botryosphaeria dothidea	8 _ 11	China	*Ficus carica*	MN428793	MN478488	MN508257
	P90	Italy: Sicily	*Pistacia vera*	MZ350238	MZ358198	MZ358263
	CBS 115476 (T)*	Switzerland	*Prunus* sp.	AY236949	AY236898	AY236927
Lasiodiplodia pseudotheobromae	CBS 116459 (T)	Costa Rica	*Gmelina arborea*	EF622077	EF622057	EU673111
	JY-1	Korea	*Ficus carica*	MH718809	MH885311	-
	ZJF-1	China	*Ficus carica*	MH127477	MH135320	MH135319
	CBS 164.96 (T)	Papua New Guinea	Fruit along coral reef coast	AY640255	AY640258	EU673110
	CPC 27885	Malta	*Citrus sinensis*	MW413871	MW419189	MW419252
Neofusicoccum algeriense	12P	Mexico	*Rubus idaeus*	KY609176	KY609174	KY609175
	CBS 137504 (T)	Algeria	*Vitis vinifera*	KJ657702	KJ657715	KX505915

	CBS 719.85	New Zealand	*Malus x domestica*	KX464151	KX464646	KX464921
	PE32	Portugal	*Eucalyptus globulus*	KT440952	KT441012	KX871765
N. australe	CMW 6853	Australia	*Sequoiadendron* sp.	AY339263	AY339271	AY339255
N. brasiliense	CMW 1338	Brazil	*Mangifera indica*	JX513630	JX513610	KC794030
N. hellenicum	P109	Italy: Sicily	*Pistacia vera*	MZ350239	MZ358199	MZ358264
N. luteum	CBS 562.92 (T)	New Zealand	*Actinidia deliciosa*	KX464170	KX464690	KX464968
N. mediterraneum	CREA-DC TPR OL.427	Italy: Apulia	*Olea europaea*	OL454501	OL539661	OL539662
	P123	Italy: Sicily	*Pistacia vera*	MZ350297	MZ358257	MZ358322
N. parvum	CBS 145622	Italy: Sicily	*Ficus carica*	MN611179	MN623346	MN623343
	CPC 28173	Italy	*Microcitrus australasica*	MW413891	MW419209	MW419272
	CMW 9081 (T)	New Zealand	*Populus nigra*	AY236943	AY236888	AY236917
N. ribis	CMW 7772 (T)	USA	*Ribes vulgare*	AY236935	AY236877	AY236906
N. vitifusiforme	CBS 110887 (T)	South Africa	*Vitis vinifera*	AY343383	AY343343	KX465061
Neoscytalidium dimidiatum	Nd_Fig01	Turkey	*Ficus carica*	OL304243	OK788657	OK788660

*: T = Ex-type strains.

Table S3. Collection details and GenBank accession numbers of *Ceratocystis* isolates included in the multi-gene phylogenetic analysis.

**Species**	**Culture No.**	**Locality**	**Host**	**GenBank accession numbers**		
				**ITS**	**TEF**	**RPB2**
Ceratocystis albifundus	CMW4068	South Africa	*Acacia mearnsii*	DQ520638	EF070400	KY644041
C. atrox	CBS 120518 (T)*	Australia	*Eucalyptus grandis*	EF070415	EF070403	KY644030
C. changhui	CBS 139797 (T)	YunNan, China	*Colocasia esculenta*	KY643884	KY643941	KY644015
C. ficicola	CMW38544	Japan	*Ficus carica*	KY685076	KY685079	KY685083
	C1355 (T)	Japan	*Ficus carica*	NR119410	KY316544	KY685082
C. fimbriata	CBS 114723 (T)	USA: North Carolina	*Ipomoea batatas*	KC493160	KJ631109	KJ601614
C. huliohia	CBS 142794 (T)	USA: Hawaii	*Metrosideros polymorpha*	KY809156	KY809118	KY809143
C. manginecans	CBS 121659 (T)	Oman	*Mangifera indica*	AY953383	EF433317	KJ601620
C. obpyriformis	CBS 122511 (T)	South Africa	*Acacia mearnsii*	EU245003	EU244935	KY644032
C. pirilliformis	CBS 118128 (T)	Australia	*Eucalyptus nitens*	AF427105	AY528983	KJ601630

C. polychroma	CBS 115778 (T)	Indonesia	*Syzygium aromaticum*	AY528970	AY528978	KY644027
C. uchidae	CBS 115164 (T)	USA: Hawaii	*Syzygium aromaticum*	AY526306	KY643921	KY643996

*: T= Ex-type strains.

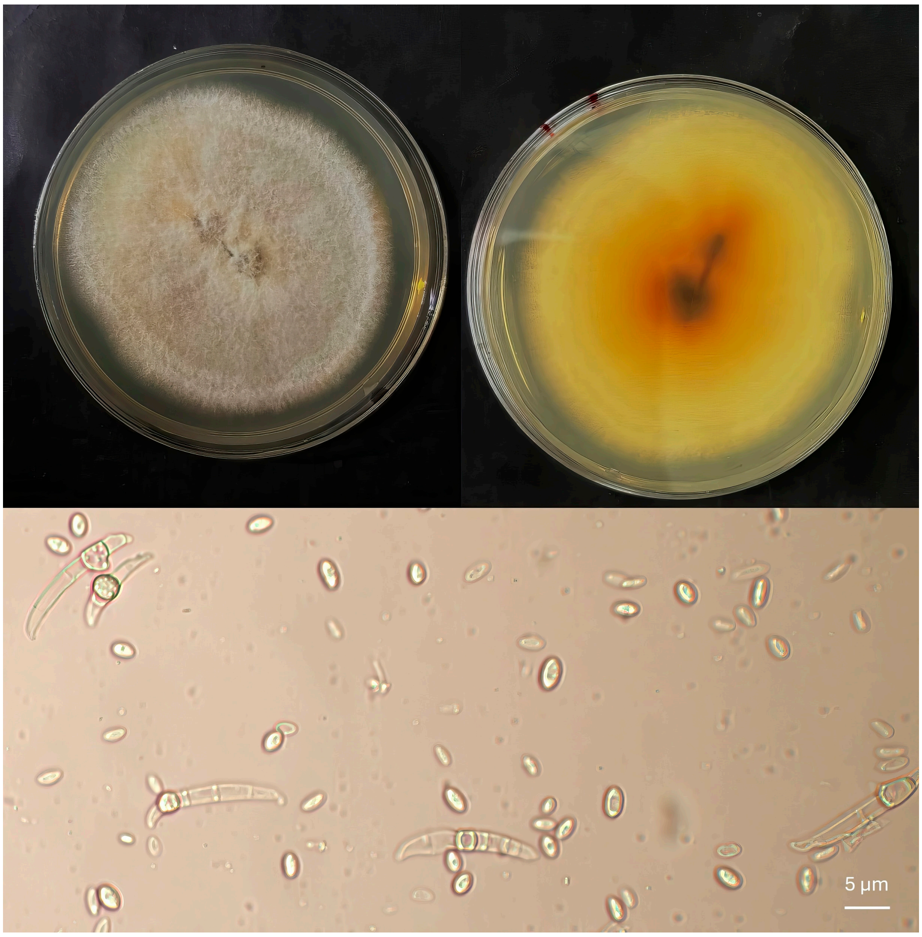

Figure S1. Morphological characteristics of *Neocosmospora perseae* CRSFA.Fus.017 isolated from symptomatic *Ficus carica* branch. Top view of the colony on potato dextrose agar (PDA) after 10 days at 25 °C, showing abundant, cottony, white mycelium (top left). Bottom view of the same colony on PDA, displaying yellow–orange pigmentation with a darker central zone (top right). Microscopic view of conidial structures, including aseptate microconidia and multi-septate falcate macroconidia, observed at 400× magnification (bottom). Scale bar = 5 µm.

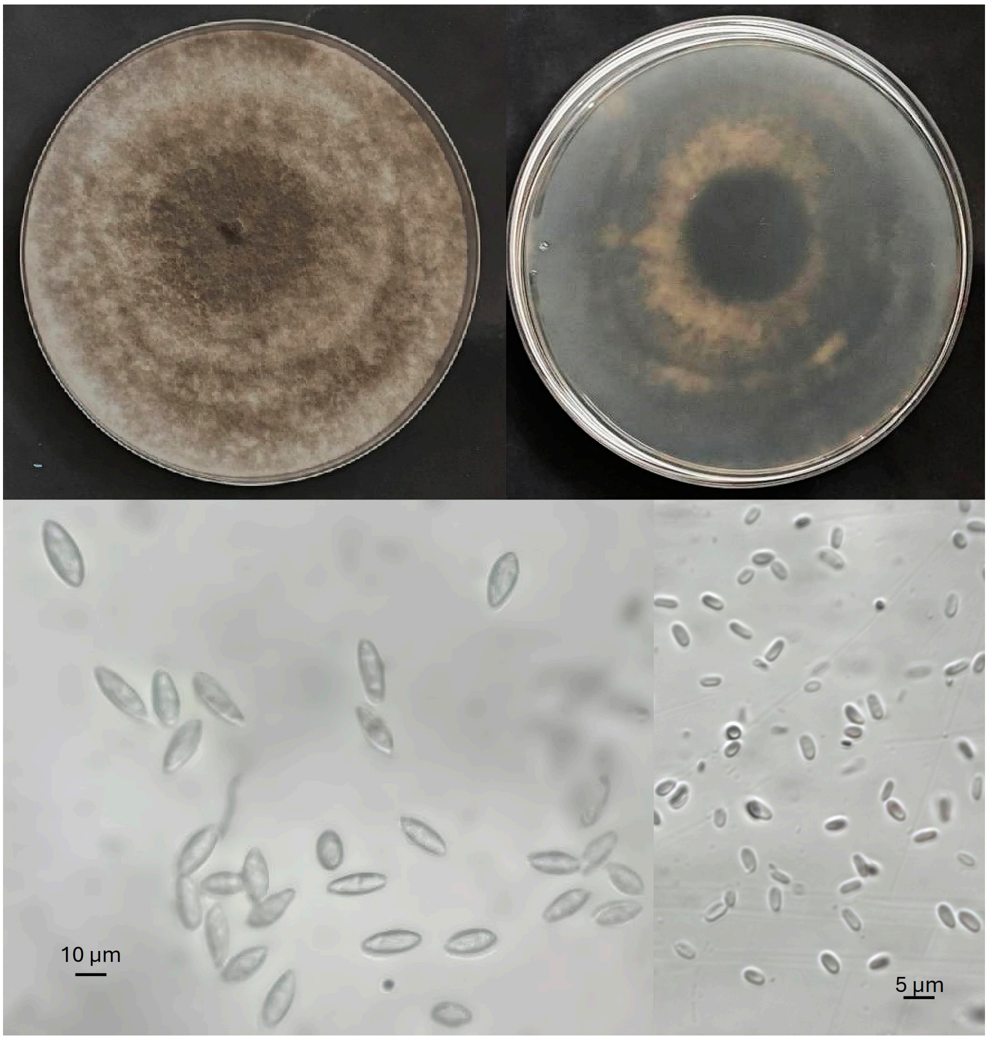

Figure S2. Morphological features of *Neofusicoccum algeriense* from symptomatic fig plant. Top (left): colony on PDA after 10 days at 25 °C (surface view); Top (right): reverse of the same colony; Bottom (left): typical hyaline, aseptate conidia (scale bar = 10 μm); Bottom (right): spermatia (scale bar = 5 μm).

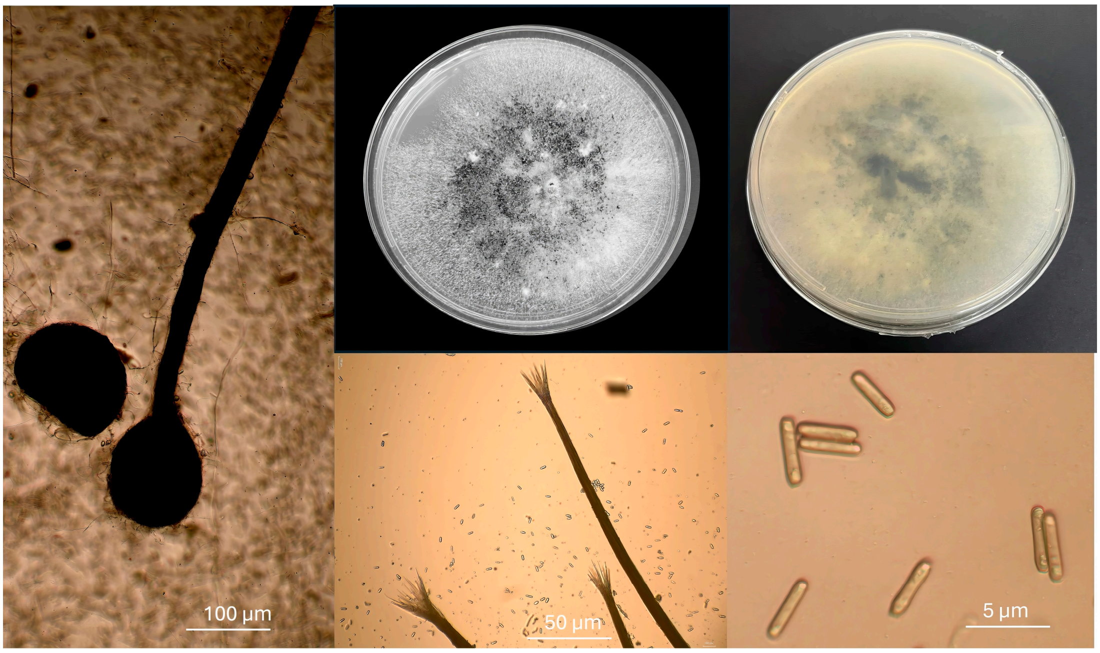

Figure S3. Morphological characteristics of *Ceratocystis ficicola* CRSFA_Cer_035. Top center: colony on PDA after 10 days at 25 °C, upper view; top right: reverse view of the same colony. Top left: perithecia developing on culture medium (scale bar = 100 μm). Bottom left: long-necked perithecia releasing cirrhi of ascospores (scale bar = 50 μm). Bottom right: typical cylindrical endoconidia observed under light microscopy (scale bar = 5 μm).
